# The effects of responsiveness, perceived warmth, and anthropomorphism on university students' use of conversational AI for learning support: a chain mediation analysis based on S-O-R framework

**DOI:** 10.3389/fpsyg.2026.1769964

**Published:** 2026-04-07

**Authors:** Yanling Lan, Sihang Liu, Hao Chen

**Affiliations:** School of Film Television and Communication, Xiamen University of Technology, Xiamen, China

**Keywords:** AI anthropomorphism, conversational artificial intelligence, human- AI interaction, learning support, Stimulus-Organism-Response framework

## Abstract

**Introduction:**

Conversational artificial intelligence (C-AI) is increasingly used by university students for learning support, yet the mechanisms through which its affective attributes shape adoption behaviors remain insufficiently understood. Drawing on the Stimulus-Organism-Response (S-O-R) framework, this study examines how AI responsiveness, anthropomorphism, and perceived warmth influence students' adoption of C-AI through AI attachment and AI trust.

**Methods:**

A cross-sectional survey was conducted among 538 Chinese university students. The proposed model tested the relationships among AI responsiveness, anthropomorphism, perceived warmth, AI attachment, AI trust, and adoption-related learning behaviors.

**Results:**

The results showed that AI responsiveness and anthropomorphism significantly strengthened students' AI attachment and AI trust, which in turn promoted their adoption of C-AI for learning support. Perceived warmth also facilitated sustained interaction and learning engagement through attachment and trust. Overall, AI attachment and AI trust served as key mediating mechanisms linking affective AI attributes to students' learning behaviors.

**Discussion:**

The findings suggest that university students' adoption of C-AI is shaped not only by technological functionality but also by emotional and relational cues embedded in AI interaction. This study extends the S-O-R framework in the context of educational AI and offers practical implications for designing human-centered, emotionally responsive, and pedagogically effective intelligent learning systems.

## 1 Introduction

As AI agents are increasingly integrated into course consultations, writing support, and personalized learning assistance, conversational artificial intelligence (Conversational AI) has become an important component of university students' daily learning practices (Malakcioglu, 2024; Tan et al., 2025). Alongside this technological expansion, the learning environment in higher education has become psychologically and socially more complex. University students commonly report elevated stress, social disconnection, and fluctuating wellbeing. Sustained academic competition and performance demands may contribute to learning burnout, academic disengagement, and reduced self-efficacy, which are recognized as significant predictors of learning behavior and psychological health (Siyou and Wan Husin, 2025). Advances in large language models (LLMs) have further enhanced the generative capacity and interactional coherence of conversational AI. As a result, conversational AI increasingly displays anthropomorphic cues, affective expressiveness, and social signals. Unlike earlier systems primarily designed for information retrieval, LLM-based conversational AI can respond to users' emotional states, strengthening contextual adaptation and affective responsiveness. Empirical evidence suggests that intelligent tools capable of detecting student emotions or providing affective support are associated with higher learning engagement and improved perceived outcomes in educational settings (Heng et al., 2025). Systems with emotion recognition and adaptive feedback functions have also been shown to identify disengagement tendencies and offer psychological support to some extent (Yaseen et al., 2025; Salloum et al., 2025). Moreover, chatbot use has been associated with reduced academic stress, enhanced self-efficacy, and lower frustration (Konadu and Kusi, 2025), and may alleviate anxiety, loneliness, and emotional distress among university students (Reyes-Portillo et al., 2025). Collectively, interaction with conversational AI is shifting from primarily information-oriented use toward a more relational and affective process. Emotional experience is increasingly embedded in learning interactions and represents an essential dimension for understanding university students' conversational AI use behavior.

When emotional experience becomes part of learning interactions, conversational artificial intelligence no longer functions only as an information tool; it also operates as a relational partner within the learning process. Research in human-computer interaction and social cognition shows that when systems display anthropomorphic cues, emotional feedback, and context-sensitive responses, users tend to apply social attribution. They may perceive the system as a quasi-social actor with intention and agency (Go and Sundar, 2019). Design features such as autonomy cues, personalization, and explainability further shape users' perceptions. These features influence users' sense of control, psychological ownership, and cognitive processing. As a result, users redefine the system's functional role and relational boundaries. With repeated interaction, users may form relatively stable affective and cognitive evaluations of the system (Shin, 2021). These evaluations can strengthen continued engagement. Shu et al. (2026) show that anthropomorphic cues facilitate emotional projection and contribute to attachment formation. Deng and Yan (2025) demonstrate that perceived warmth enhances emotional security and relational trust. Wang et al. (2026) further find that timely and coherent responsiveness increases social presence and perceived reliability. This process reinforces trust in the system's competence and stability. Through these mechanisms, interaction may gradually shift from task-oriented cooperation to relationship-oriented engagement. However, relational intensification has both benefits and risks. High-quality interaction may increase learning engagement and support emotional regulation. At the same time, excessive anthropomorphism and emotionally intensive interaction may lead to cognitive offloading, reduced autonomy, and blurred relational boundaries. In high-frequency contexts, the system may become integrated into users' cognitive and emotional routines. This integration can reshape how users evaluate technological reliability and relational stability (Alqasir, 2025). Therefore, anthropomorphic cues, perceived warmth, and responsiveness are central interaction features. They shape perceptions of the system's social attributes and provide the psychological basis for the development of attachment and trust. Examining these mechanisms is essential for understanding how AI usage behavior develops among university students.

As human-AI relationships evolve and affective processes gain prominence, prior research has offered important insights into conversational artificial intelligence. Nevertheless, several theoretical issues remain unresolved. First, from a model integration perspective, existing studies have examined anthropomorphic cues, perceived warmth, attachment, and trust in relation to use behavior. However, these constructs are typically tested through separate paths or treated as supplementary effects. Few studies clearly explain how interaction cues influence behavior through relational states within a unified theoretical framework. As a result, the structural connection among external stimuli, relational cognition, and behavioral outcomes is not yet clearly specified. Second, affective variables are often positioned as peripheral components within technology adoption models rather than as central explanatory mechanisms. Although anthropomorphic expression and emotional responsiveness are increasingly recognized, they are commonly framed as enhancements to cognitive evaluation rather than as independent affective drivers of engagement. This positioning limits the explanatory role of emotional experience in sustained human-AI interaction. Third, higher education is characterized by frequent interaction and high cognitive demands. Students face distinct motivational pressures and stressful conditions. Yet, empirical research has not sufficiently incorporated these contextual features into analyses of conversational artificial intelligence use. As students repeatedly rely on such systems for course assistance and writing support, interaction experiences may gradually consolidate into stable relational judgments. The theoretical logic underlying this transition remains insufficiently articulated. Therefore, integrating interaction cues, relational states, and use behavior within a coherent structural framework is essential for advancing the understanding of affective engagement mechanisms in higher education.

Accordingly, this study examines key affective features of conversational artificial intelligence—anthropomorphism, emotional expressiveness, and perceived realism—to understand how university students engage with AI in learning contexts. Drawing on the Stimulus-Organism-Response (S-O-R) framework, AI responsiveness, anthropomorphic cues, and perceived warmth are conceptualized as external stimuli. These stimuli shape internal relational states, namely AI attachment and AI trust, which subsequently influence AI use behavior. This approach specifies a three-stage structure linking technological stimuli, relational experience, and behavioral outcomes. Partial least squares structural equation modeling (PLS-SEM) is employed to test the direct and indirect relationships among stimuli, relational states, and behavior, including the sequential mediating roles of attachment and trust. By integrating psychological mechanisms with interaction design features, the study provides a structured account of affective engagement in AI-supported higher education. The findings contribute to instructional design and inform the sustainable integration of conversational AI in learning environments.

## 2 Literature review

### 2.1 Conversational AI in higher education from interactive support to emotional engagement

Conversational artificial intelligence (conversational AI), commonly referred to as chatbots, enables human-computer interaction through natural language processing (NLP) technologies (Bradeško and Mladenić, 2012). Since the early development of ELIZA, which relied on pattern-matching techniques to simulate dialogue (Weizenbaum, 1966), conversational AI has evolved from text-based systems into interactive agents capable of multimodal communication and contextual feedback (Dehn and Van Mulken, 2000; Wik and Hjalmarsson, 2009). With the advancement of generative models, these systems demonstrate increased responsiveness and interactional flexibility, allowing students to engage in continuous dialogue and receive real-time feedback. Such responsiveness has been identified as a key antecedent of emotional experience in human-AI interaction (Winkler and Söllner, 2018). In higher education settings, conversational AI has been widely applied to programming instruction, language learning, and personalized academic support (Kocdar, 2017). Rather than replacing instructors, it functions as an interactive learning assistant that provides immediate guidance, process-oriented feedback, and task-related scaffolding (Daud et al., 2020). During sustained interaction between university students and conversational AI, learning experiences gradually extend beyond task-oriented support toward emotional and relational perceptions. Prior research indicates that anthropomorphic cues, including human-like language, embodied interaction signals, and perceived warmth, encourage users to interpret conversational AI as a social actor, thereby increasing emotional engagement and psychological involvement (Schouten et al., 2023; Donnermann et al., 2025).

Such emotional experiences can reshape usage patterns. When interactions are perceived as coherent and supportive, students are more likely to seek ongoing feedback and sustain participation (Lee et al., 2023). Beyond evaluations of informational usefulness, users gradually form affect-based judgments about conversational AI, which influence relational engagement and continued interaction. Through repeated exposure and accumulated emotional responses, conversational AI may become embedded in students' daily learning and emotion-regulation practices, fostering relational bonding conceptualized as AI attachment (Laban et al., 2025). Consistent emotional responsiveness further strengthens long-term engagement and behavioral persistence (Feng et al., 2022). Accordingly, within higher education contexts, conversational AI interaction is increasingly shifting from task-oriented assistance toward emotionally grounded engagement (Alam, 2022). As conversational AI assumes these relational functions, understanding how such emotional engagement develops becomes theoretically necessary. Although prior research links anthropomorphic and responsive cues to enhanced engagement, the affective mechanisms through which technological cues translate into attachment, trust, and sustained AI use remain insufficiently integrated. Therefore, a systematic examination of emotional perception in human-conversational AI interaction is warranted.

### 2.2 Mechanisms of emotional engagement in human-conversational AI interaction

In real-world human-AI interaction contexts, conversational artificial intelligence increasingly participates in users' social communication through text- and voice-based natural language exchanges. Its human-like expression and high interaction fidelity encourage users to perceive it as a socially responsive agent rather than a purely technical tool (Akpan et al., 2025). This anthropomorphic tendency is reflected across contemporary conversational AI systems. For instance, embodied robots such as Pepper integrate generative models to enable contextual dialogue and voice interaction, prompting users to interpret them as intentional social actors (Bertacchini et al., 2023). Similarly, multimodal systems such as Claude and Gemini, as well as avatar-based tools like Doubao in the Chinese context, enhance social presence and emotional accessibility through human-like language styles and virtual representations (Haman et al., 2024). These applications indicate that AI anthropomorphism operates not only as a design feature but also as a social cue that activates emotional processing and relational interpretation (Rao Hill and Troshani, 2024). When users perceive warmth and empathic signals during interaction, AI-perceived warmth can reduce uncertainty and increase emotional security, facilitating early affective engagement (Seitz, 2024). With repeated interaction, emotional experiences associated with anthropomorphic cues and perceived warmth can develop into AI attachment. This process reshapes participation patterns and increases usage frequency as conversational AI shifts from a functional tool to a relational partner. Evidence from avatar-based and immersive interaction environments further shows that high realism and social cues may strengthen emotional involvement while reducing cognitive load, promoting sustained use behavior (Fink et al., 2024; Veras et al., 2023; Liu and Liu, 2025). Consequently, when conversational AI is framed as a collaborative partner or emotional companion, users tend to demonstrate stronger relational engagement and higher interaction frequency, although attachment-driven engagement may also introduce risks of emotional dependency and adhesive use patterns (Kyrlitsias and Michael-Grigoriou, 2022). Such attachment, reinforced through real interaction experiences, not only shapes conversational AI use behavior but also provides a contextual foundation for the subsequent development of trust.

Humans increasingly perceive conversational AI as socially embodied agents, largely due to anthropomorphism, which activates social cognition and emotional responses during human-AI interaction (Seitz, 2024). Through human-like language styles, responsive dialogue, and virtual avatars, conversational AI is often interpreted as a relational partner rather than a purely instrumental tool (Anisha et al., 2024). Anthropomorphic cues enhance perceived warmth and relational closeness, fostering early emotional engagement even in the initial stages of interaction. In contrast to traditional technology models that position trust as a prerequisite for attachment, anthropomorphic conversational AI may evoke affective bonding before stable cognitive evaluations are formed (Fink et al., 2024). Because social cues are often processed heuristically, high responsiveness and interaction fidelity can reduce uncertainty and negative emotions, allowing users to develop attachment based on experiential interaction rather than verified system competence (Veras et al., 2023). This affect-driven process suggests that attachment may function as a precursor to trust, as relational identification and emotional investment gradually evolve into cognitive confidence in the system. When conversational AI is framed as a collaborative partner or emotional companion, users are more likely to internalize the interaction as socially meaningful, strengthening attachment and facilitating trust development despite limited technical knowledge. However, attachment-driven engagement may also increase emotional dependency and high-frequency adhesive use, highlighting the dual-edged nature of anthropomorphism in conversational AI interaction (Waytz et al., 2014; Kyrlitsias and Michael-Grigoriou, 2022).

## 3 Theoretical framework and research hypothesis

### 3.1 Stimulus-Organism-Response model (S-O-R)

Stimulus-Organism-Response (S-O-R) theory, originally proposed by Mehrabian and Russell (1974), conceptualizes behavior as a process in which external stimuli influence individuals' internal states, which subsequently shape behavioral responses. Developed to extend the traditional stimulus-response (S-R) model, S-O-R emphasizes the mediating role of internal emotional processes between environmental cues and behavioral outcomes (Hochreiter et al., 2023). The framework consists of three core components: stimulus (S), referring to external environmental or technological cues perceived by individuals; organism (O), representing internal cognitive and emotional states triggered by these stimuli; and response (R), denoting behavioral intentions or actual actions (Jacoby, 2002; Wang et al., 2023). Beyond its origins in environmental psychology, S-O-R has evolved into a widely adopted analytical framework in digital and intelligent contexts. Prior studies have applied S-O-R to examine impulse buying in live-streaming commerce (Huo et al., 2023), information overload and misinformation sharing in social media environments (Apuke et al., 2024), and health information avoidance behavior among Generation Z (Jia and Li, 2024). In the field of human-computer interaction, S-O-R has been used to explain how technological affordances, interactivity, and trust-related cues influence user engagement and behavioral intention (Shi et al., 2022). These applications demonstrate the framework's flexibility in capturing how multiple external stimuli within digital environments evoke emotional and cognitive responses that ultimately guide user behavior. Accordingly, S-O-R provides a robust theoretical foundation for examining emerging technology adoption across diverse scenarios (Huang, 2023).

Compared with the Technology Acceptance Model (TAM), which emphasizes perceived usefulness and perceived ease of use as determinants of usage behavior (Davis, 1989), and the Unified Theory of Acceptance and Use of Technology (UTAUT), which explains technology adoption through performance expectancy and social influence (Venkatesh et al., 2003), these frameworks primarily rely on rational evaluation. Their focus on instrumental cognition provides a limited explanation of affective processes such as emotional perception, trust, attachment, and relational dynamics in human-AI interaction. Likewise, the Theory of Planned Behavior (TPB), the Expectation-Confirmation Model of Information Systems Continuance (ECM-IS), and Social Cognitive Theory (SCT) remain centered on performance beliefs, expectations, and social norms, offering comparatively limited insight into the emotional fluctuations and relational experiences emerging during technology use (Ajzen, 1991; Bhattacherjee, 2001). By contrast, the Stimulus-Organism-Response (S-O-R) framework explicitly incorporates affective mediation into its structure. It conceptualizes external cues as stimuli, internal emotional and cognitive states as organism-level processes, and behavioral outcomes as responses, making it particularly suitable for examining conversational AI in intelligent education contexts. As students increasingly perceive conversational AI as a learning partner or companion, affective mechanisms become central to engagement. At the stimulus level, S-O-R accommodates multidimensional technological characteristics and social cues; at the organism level, it incorporates emotional experience, perceived warmth, and psychological attachment. This structure moves beyond the linear “external driver–cognition–behavior” logic and enables analysis of how conversational AI cues evoke emotional perception and relational attachment, thereby influencing university students' AI-assisted learning behavior (Vafaei-Zadeh et al., 2025). Recent studies have applied the S-O-R model in intelligent education and human-computer interaction research (Yang et al., 2021; Peng et al., 2023; Duong, 2024). Building on this literature, the present study conceptualizes AI perceived warmth, AI anthropomorphism, and AI responsiveness as stimulus-level variables, as they represent socially embedded technological cues in human-AI interaction. AI attachment and AI trust are specified as organism-level variables, reflecting affective and relational states formed during interaction (Hu et al., 2021). Accordingly, this study examines how responsiveness, perceived warmth, and anthropomorphism shape university students' attachment and trust, thereby influencing AI-assisted learning behavior. This approach provides an integrated account of the pathway linking technological characteristics, affective mechanisms, and learning behavior in intelligent education contexts.

### 3.2 Research hypothesis

#### 3.2.1 AI responsiveness

Previous studies have demonstrated that, across diverse human-computer interaction contexts, system responsiveness is positively associated with users' emotional responses and cognitive evaluations. In the context of conversational AI supporting university students' learning processes, responsiveness is not only regarded as a technical attribute but also as a key source of content-based responses and affective feedback that reinforces learners' feelings of being attended to, responded to, and understood (Chen and DiFranzo, 2025). When conversational AI agents fail to respond to students' needs in a timely manner, users may experience frustration or disappointment, which in turn weakens their frequency of technology use and attachment behaviors, ultimately leading to negative outcomes such as distrust and non-use (Zhang R. W. et al., 2024). Conversely, when conversational AI is able to respond to students' needs and questions in an immediate, accurate and timely manner, such real-time interaction and feedback can effectively enhance students' instant gratification, wellbeing, and sense of identification. This form of positive human-AI interaction not only facilitates the development of emotional attachment among learners but also strengthens their judgments regarding the reliability and trustworthiness of AI agents (Al-Oraini, 2025). Therefore, in intelligent education contexts, conversational AI with high responsiveness is more likely to enhance learners' attachment to and trust in AI. Based on this reasoning, the present study proposes the following hypotheses:

Hypothesis 1: AI responsiveness is positively associated with university students' AI attachment toward conversational AI.
Hypothesis 2: AI responsiveness is positively associated with university students' AI trust toward conversational AI.

#### 3.2.2 AI Anthropomorphism

AI Anthropomorphism is commonly conceptualized as comprising appearance-based anthropomorphism and behavior-based anthropomorphism. This anthropomorphic characteristic plays a critical role in explaining perceived emotions and cognitive evaluations in human-AI interaction (Kim and Sundar, 2012). Klaus and Zaichkowsky (2020) further emphasized that anthropomorphism can effectively support users' experiential engagement and trust when using conversational AI as learning tools. Sheehan et al. (2020) argued that the anthropomorphic features of conversational AI can satisfy students' social needs, create positive interaction experiences, and, in some cases, even promote continuance or subscription-related behaviors. In intelligent education contexts, conversational AI endowed with anthropomorphic characteristics exhibits more human-like attributes, potentially altering attribution dynamics when system errors or contradictions occur and fostering interaction patterns that resemble human-human communication. As a result, students tend to develop stronger emotional connections and psychological attachment toward conversational AI agents driven by large language models (LLMs) (Shahzad et al., 2024). Moreover, conversational AI that displays stronger anthropomorphic traits can enhance users' perceptions and evaluations of its capabilities, thereby further facilitating the formation and stabilization of trust (Chi and Hoang, 2023). Based on these arguments, the present study proposes the following hypotheses:

H3: AI anthropomorphism is positively associated with university students' AI attachment toward conversational AI.
H4: AI anthropomorphism is positively associated with university students' AI trust toward conversational AI.

#### 3.2.3 AI Perceived warmth

AI perceived warmth refers to the extent to which students perceive “warmth” and “care” during their interactions with conversational AI. This perception is typically shaped by multiple factors, including the AI's response speed, tone, and linguistic style (McKee et al., 2023). Prior studies have indicated that when responses generated by conversational AI contain rich affective cues and considerate content, users tend to experience higher levels of emotional satisfaction and affinity, which in turn facilitates the formation of emotional attachment (Skjuve et al., 2021). Reeves et al. (2020) further found that the enthusiasm and perceived warmth of conversational AI constitute important determinants of human-AI interactivity and attachment, underscoring the importance of content-level design in conversational AI agents. Mieczkowski et al. (2019) extended this line of research by examining the relationships among perceived warmth, competence, emotional responsiveness, and user behaviors across diverse application contexts of conversational AI. As an external stimulus at the level of technological feedback, AI perceived warmth can effectively strengthen students' emotional connection and attachment to conversational AI. Moreover, AI perceived warmth not only influences students' emotional attachment but also plays a critical role in the establishment of trust. Conversational AI agents with higher perceived warmth are more likely to provide users with a sense of security and reliability, thereby enhancing students' trust in AI agents (Chi and Hoang, 2023). Based on these arguments, the present study proposes the following hypotheses:

H5: AI perceived warmth is positively associated with university students' AI attachment toward conversational AI.
H6: AI perceived warmth is positively associated with university students' trust in conversational AI.

#### 3.2.4 AI attachment

AI attachment refers to the emotional bond that users develop through repeated interactions with conversational AI. Anthropomorphic conversational agents, through human-like communication and relational interaction patterns, can evoke social responses that encourage users to perceive AI as a social partner rather than a purely functional system (Deng and Yan, 2025). While traditional trust theories often position trust as a prerequisite for attachment, this sequence may differ in conversational AI contexts. Because ongoing dialogue and anthropomorphic cues activate social heuristics, users may form attachment based on interaction experiences before engaging in deliberate cognitive evaluations of system reliability (Go and Sundar, 2019). In such interactions, attachment can function as an affective precursor that shapes subsequent cognitive trust by increasing users' willingness to rely on AI recommendations (Qiu and Benbasat, 2009). Empirical studies further indicate that attachment toward AI tools can facilitate trust formation through experience-based relational mechanisms. Sharpe and Ciriello (2024) showed that different forms of user attachment shape how individuals interpret AI reliability and sustain reliance over time, allowing trust to emerge from continued interaction rather than prior evaluation. Youn and Jin (2021) similarly demonstrated that emotional bonding increases positive trust judgments by encouraging deeper and more frequent engagement, which gradually stabilizes users' expectations of AI performance. In companion-oriented conversational AI contexts, Chaturvedi et al. (2023) found that perceived psychological closeness promotes ongoing involvement with the AI agent, enabling trust to develop as a cognitive consolidation of repeated relational experience. Accordingly, particularly in anthropomorphic conversational AI environments, emotional attachment may plausibly precede and facilitate cognitive trust rather than merely result from it. Based on this reasoning, the present study proposes the following hypotheses:

H7: AI attachment is positively associated with university students' AI trust toward conversational AI.
H8: AI attachment is positively associated with university students' AI usage behavior related to conversational AI-assisted learning.

AI attachment is regarded as a key mechanism through which affective and social factors mediate behavior in intelligent education contexts. Specifically, AI responsiveness, AI anthropomorphism, and AI perceived warmth constitute critical antecedents that strengthen users' emotional attachment. Empirical evidence suggests that AI responsiveness enhances students' emotional engagement with intelligent systems (Nguyen and Le, 2025), whereas anthropomorphism and perceived warmth foster affinity and attachment, thereby reinforcing students' emotional connections with AI (Ma et al., 2025). Munnukka et al. (2022), focusing on AI-based customer service systems, found that frequent interaction with AI agents positively influences users through responsiveness and anthropomorphic features, leading to attachment formation and subsequently promoting AI usage behavior. More recent research by Nikolov et al. (2025) further indicated that when users perceive anthropomorphic characteristics and convenience benefits in AI agents, their usage frequency and attachment tendencies increase significantly, thereby enhancing AI usage behavior across diverse contexts. Accordingly, the present study proposes the following hypothesis:

H9: AI attachment plays a significant mediating role in the relationships between AI responsiveness, AI Anthropomorphism, AI perceived Warmth, and AI usage behavior.

#### 3.2.5 AI trust

Existing research indicates that trust in conversational AI is not only a core component of users' decision-making processes but also directly influences their usage behavior (Alagarsamy and Mehrolia, 2023). Users with higher levels of trust tend to engage more actively in interactions and respond more positively to feedback provided by AI agents. Particularly in learning contexts, increased AI trust facilitates the development of students' long-term reliance on AI, thereby promoting sustained use and enhancing learning motivation. In contrast, when students lack trust in conversational AI agents, they are more likely to avoid these systems and reduce their reliance on them, which in turn negatively affects their usage behavior (Seo, 2022). Based on this reasoning, the present study proposes the following hypothesis:

H10: AI trust is positively associated with university students' AI usage behavior related to conversational AI-assisted learning.

In addition, the responsiveness of conversational AI can enhance students' trust in the system by providing timely and accurate feedback, thereby stimulating their motivation to use AI tools (Zhang X. et al., 2024). Meanwhile, the anthropomorphic characteristics of conversational AI enable students to perceive human-like interaction qualities, and such humanized interaction patterns can strengthen students' trust in conversational AI, further promoting their usage behavior (Li et al., 2023). AI perceived warmth—such as friendliness, care, and positive interactive content—can also increase students' dependence on and trust in conversational AI. When students develop trust in conversational AI, they are not only more willing to rely on these systems to support their learning but also tend to increase their usage frequency and improve their academic efficacy and task completion levels (Romero-Charneco et al., 2025). Accordingly, the present study proposes the following hypothesis:

H11: AI trust plays a significant mediating role in the relationships between AI responsiveness, AI Anthropomorphism, AI perceived warmth, and AI usage behavior.

#### 3.2.6 Chain mediation effects of AI attachment and AI trust

**Figure 1:**
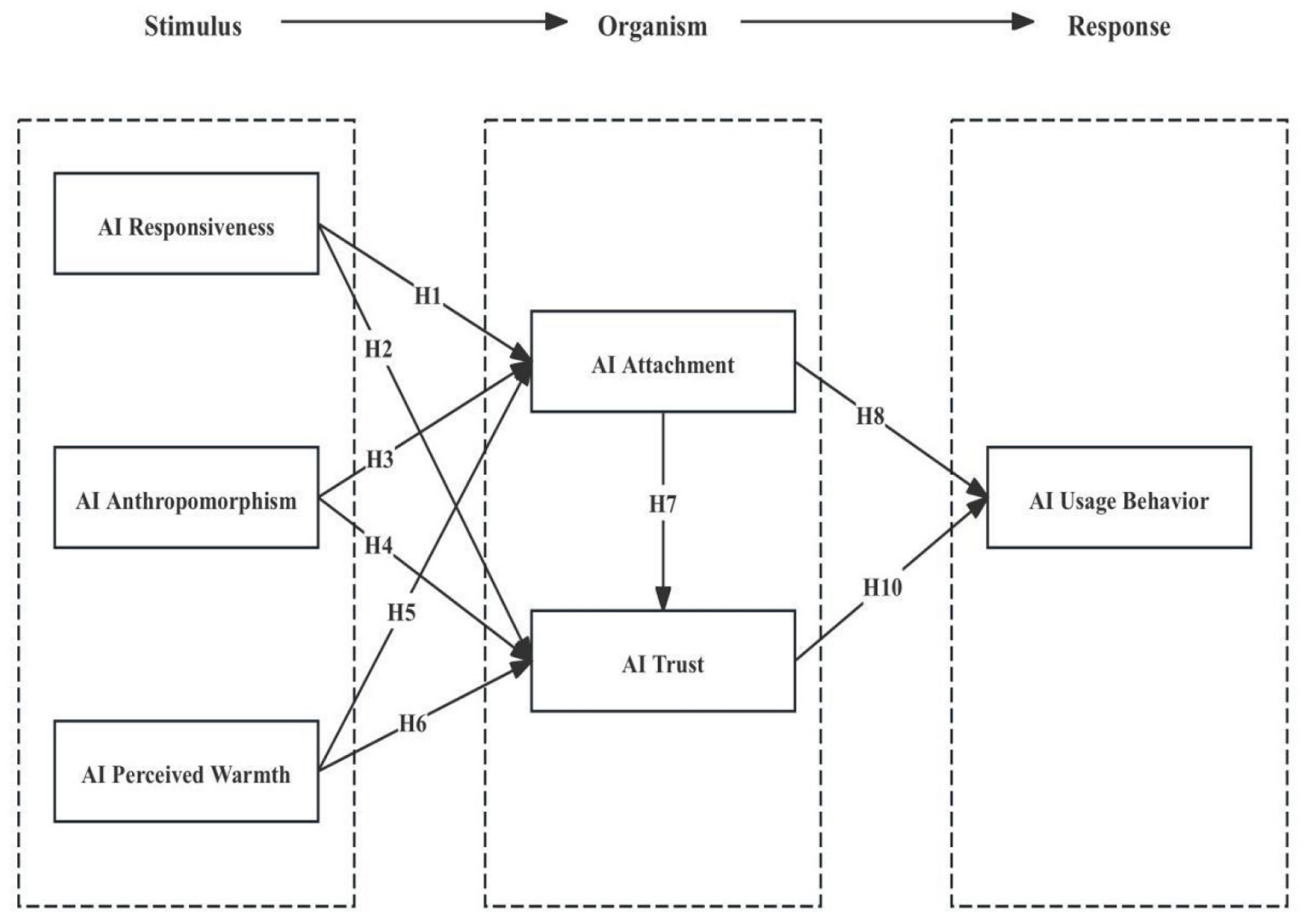
Structural equation modeling framework based on S-O-R.

Existing studies have demonstrated that anthropomorphic design, response speed, and perceived warmth of AI agents can enhance students' trust in AI by strengthening their emotional connection with these systems (Wut et al., 2025). As external stimuli, technologically driven support mechanisms exert direct influences on users' experiential accumulation and emotional perceptions. Through information retrieval, emotional companionship, and interactive experiences provided by conversational AI, users can obtain attachment-related gratification, enhance individual pleasure and wellbeing, and further increase their trust in and demand for AI technologies (Alagarsamy and Mehrolia, 2023). Trust can reduce students' concerns regarding system malfunctions, potential risks, and algorithmic bias, thereby motivating them to adopt conversational AI in learning contexts (Pitts and Motamedi, 2025). Ding and Najaf (2024) verified that the interactivity and humanness of online conversational AI agents significantly increase users' interaction frequency and attachment, strengthen technological trust, and improve usage levels. Dang and Li (2025) further argued that trust and uncertainty are key moderating factors influencing the diffusion of AI technologies. High levels of trust generated through frequent and intimate interactions can mitigate concerns related to potential bias, uncertainty, and safety risks, ultimately promoting technology acceptance and usage behavior. More specifically, functional technological support intensifies human-AI interaction frequency and fosters attachment formation. AI attachment not only influences students' trust in AI but also affects their usage frequency and willingness to engage in sustained interaction. Based on this reasoning, the present study proposes the following hypothesis. The specific theoretical model is shown in Figure 1.

H12: AI attachment and AI trust jointly exert a significant chain mediating effect in the relationship between AI responsiveness and AI usage behavior.
H13: AI attachment and AI trust jointly exert a significant chain mediating effect in the relationship between AI anthropomorphism and AI usage behavior.
H14: AI attachment and AI trust jointly exert a significant chain mediating effect in the relationship between AI perceived warmth and AI usage behavior.

## 4 Materials and methods

### 4.1 Participants

This study employed a cross-sectional survey design to examine the structural relationships among AI responsiveness, AI anthropomorphism, AI perceived warmth, AI attachment, AI trust, and AI use behavior among Chinese university students interacting with conversational artificial intelligence. Prior to data collection, an a priori statistical power analysis was conducted using G^*^Power (Faul et al., 2009) to determine the minimum required sample size for regression-based path analysis. Following Cohen (1988) guidelines, a medium effect size (*f*^2^ = 0.15), a Type I error rate of 0.05, a statistical power of 0.80, and five predictor variables were specified. The analysis indicated that at least 92 participants were required to achieve adequate statistical sensitivity. Considering the practical context of online data collection, the survey was distributed through online platforms (Wenjuanxing and WeChat), and participants were recruited using a non-probability convenience sampling approach. Recruitment efforts aimed to reach students from diverse academic majors and grade levels in order to obtain a heterogeneous sample of university students. Online dissemination enabled broad access to potential participants and facilitated efficient data collection across multiple student subgroups.

Data were collected using structured questionnaires administered via the Wenjuanxing platform. The questionnaire was designed to capture multiple theoretically grounded constructs, including AI responsiveness, AI perceived Warmth, AI anthropomorphism, and AI trust, among other relevant variables. The survey was distributed through online media platforms (Wenjuanxing, WeChat). Data collection took place from November to December, 2025 and lasted for 1 month to ensure accessibility across different levels of digital literacy and technological proficiency. All participants provided informed consent prior to completing the questionnaire. A total of 565 questionnaires were distributed. Based on predefined exclusion criteria, invalid responses were identified and removed if they met either of the following conditions: (1) missing responses to key questionnaire items, or (2) a response time shorter than the average completion time (i.e., less than 1 min). After excluding 27 invalid questionnaires, the final response rate was 95.2%. The final valid sample consisted of 538 responses (*N* = 538). The collected data were imported into SPSS 26 for descriptive and demographic analysis. SmartPLS (version 4.1) was used to evaluate the proposed research model, including the relationships among latent variables and the structural paths specified in the model.

### 4.2 Participants and sampling

Participants in this study were undergraduate students recruited from higher education institutions in Fujian Province, China, through online dissemination using a non-probability convenience sampling approach. The final sample consisted of 40.5% male and 59.5% female students. In terms of age and academic standing, third-year students accounted for the largest proportion of the sample (44.1%), corresponding to 249 participants. Regarding the duration of conversational AI use, the largest proportion of participants reported using conversational AI tools for approximately 2 years (*n* = 201), whereas the smallest proportion reported a usage duration of less than 1 year (*n* = 73). In addition, with respect to usage frequency, the most common pattern was using conversational AI tools several times per week, reported by 277 participants. Detailed demographic characteristics of the sample are presented in Table 1.

**Table 1:** Frequency analysis of demographic variables.

Variables	Options	Percentage	Average	SD
Gender	Male	40.50%	1.59	0.241
	Female	59.5		
Grade	Freshman year	7.80%	2.53	0.569
	Soph more year	40.00%		
	Junior year of college	44.10%		
	Senior year of college	8.10%		
Duration of usage	than 1 year	12.90%	2.57	0.845
	1 year	34.50%		
	2 years	35.60%		
	More than 3 years	17.00%		
Frequency of usage	A few times a day	35%	1.89	0.732
	A few times a week	49%		
	Once a week	8.10%		
	Less than once a week	7.80%		

### 4.3 Measures

All constructs in this study were measured using multi-item Likert-type scales. The measurement instruments were adapted from previously validated and well-established scales and were further modified to align with the Chinese contextual setting and the intelligent education environment. Unless otherwise specified, all scale items were assessed using a five-point Likert scale ranging from 1 (“strongly disagree”) to 5 (“strongly agree”). To ensure content validity, all measurement items were reviewed by subject-matter experts in the relevant field. During the translation process, a translation and back-translation procedure was conducted by two doctoral students and three master's students. Prior to full-scale data collection, a pilot study was conducted with 50 eligible participants to assess the clarity and reliability of the questionnaire, thereby ensuring the overall comprehensibility and measurement quality of the survey instrument.

#### 4.3.1 AI responsiveness

The AI responsiveness scale was adapted from Liu (2003) and subsequent conversational AI research (Kang et al., 2024) to assess students' perceptions of interaction efficiency, feedback timeliness, and task-oriented support. To align with the educational technology context, items were contextually modified to reflect university students' experiences in AI-assisted learning. All items were measured using a five-point Likert scale. The construct demonstrated excellent internal consistency (Cronbach's α = 0.925). Detailed measurement items are presented in Table 2.

**Table 2:** Reliability and validity analysis of questionnaire.

Items	Cronbach's alpha	KMO
AI responsiveness	0.925	0.968
AI perceived warmth	0.893	
AI anthropomorphism	0.879	
AI trust	0.874	
AI attachment	0.883	
AI usage behavior	0.813	

#### 4.3.2 AI anthropomorphism

The AI anthropomorphism scale was adapted from Bartneck et al. (2009) and later applications in conversational AI research (Lee et al., 2023) to assess students' perceptions of humanlike characteristics and social presence in human-AI interaction. Items were contextually refined to reflect conversational AI–assisted learning scenarios. Responses were measured using a five-point Likert scale (1 = strongly disagree, 5 = strongly agree). The construct demonstrated satisfactory internal consistency (Cronbach's α = 0.879).

#### 4.3.3 AI Perceived warmth

The AI perceived warmth scale was adapted from the Robotic Social Attributes Scale (Carpinella et al., 2017) and subsequent conversational AI research (Zhang et al., 2025) to assess students' perceptions of warmth, care, and emotional support during human-AI interaction. Items were contextually modified to reflect AI-assisted learning scenarios in higher education. Responses were measured using a five-point Likert scale. The construct demonstrated good internal consistency (Cronbach's α = 0.893).

#### 4.3.4 AI attachment

The AI attachment scale was adapted from attachment-based measures extended to human-AI interaction contexts (Yang and Oshio, 2025), drawing on the Experiences in Close Relationships framework (Brennan et al., 1998) to assess students' emotional bonding and perceived relational closeness with conversational AI. Items were contextually refined to reflect AI-assisted learning interactions in higher education. Responses were measured using a five-point Likert scale. The construct demonstrated good internal consistency (Cronbach's α = 0.883).

#### 4.3.5 AI trust

The AI trust scale was adapted from established technology trust frameworks (McKnight et al., 1998; Hoff and Bashir, 2015) and later applications in human-AI service contexts (Flavián and Casaló, 2021) to assess students' perceptions of reliability, integrity, and trustworthiness in conversational AI systems. Items were contextually refined to reflect AI-assisted learning environments. Responses were measured using a five-point Likert scale. The construct demonstrated good internal consistency (Cronbach's α = 0.874).

#### 4.3.6 AI usage behavior

The AI usage behavior scale was adapted from prior AI adoption research (Wang and Chen, 2024) to assess students' actual engagement with conversational AI in learning contexts. The construct was measured using four items on a five-point Likert scale (1 = strongly disagree, 5 = strongly agree). The scale demonstrated acceptable internal consistency (Cronbach's α = 0.813).

### 4.4 Ethical declarations

This study involved human participants, and all procedures complied with institutional and international ethical standards for research involving human subjects. The study was conducted in accordance with the Declaration of Helsinki and was reviewed and approved by the Ethics Committee of the School of Film and Communication at Xiamen University of Technology (Approval Number: Xut-sfc-2025-07) prior to data collection. Participants were informed of the study objectives, the voluntary nature of participation, confidentiality protection, and their right to withdraw at any time without penalty. Electronic informed consent was obtained from all participants before completing the survey. All data were collected anonymously and used solely for academic research purposes.

## 5 Data analysis

Descriptive statistical analysis was conducted using SPSS version 29. SmartPLS (version 4.1) was employed to assess the reliability and discriminant validity of the measurement model and to empirically test the structural equation model proposed in this study. Statistical significance levels were set at *p* < 0.05, *p* < 0.01, and *p* < 0.001. Bootstrapping procedures implemented in SmartPLS were used to examine direct effects, indirect effects, and total effects.

SmartPLS (version 4.1) was further utilized to evaluate the theoretical model constructed in this study. Partial Least Squares Structural Equation Modeling (PLS-SEM) has been widely applied across multiple disciplines—including education, economics, and computer science—to assess large and complex models (Hair et al., 2021a). PLS-SEM follows a causal-predictive paradigm and is specifically designed to test the predictive capability of models carefully developed based on theory and logical reasoning (Sarstedt et al., 2022). Owing to its strong predictive performance, PLS-SEM not only enables path estimation based on sample data but also supports further predictive analysis through composite latent variable scores generated by the model (Hair et al., 2019). Compared with other structural equation modeling techniques, PLS-SEM demonstrates greater predictive accuracy and stronger statistical capability in analyzing emerging technology-related factors (Donath et al., 2024).

To rigorously assess the absence of potential common method variance (CMV) in this study, two methodological approaches were employed. First, variance inflation factors (VIFs) were calculated to evaluate potential multicollinearity within the model. All VIF values for the constructs in the inner model were below the recommended threshold of 3.3, with the lowest value being 1.335 (see Table 3), indicating that CMV was not a concern in the present study (Kock, 2015).

**Table 3:** Evaluation of convergent validity.

Variable	Items	FL	CA	CR	VIF	AVE
AI responsiveness	AIR1	0.852	0.925	0.941	2.69	0.727
	AIR2	0.857			2.723	
	AIR3	0.837			2.4	
	AIR4	0.853			2.649	
	AIR5	0.85			2.66	
	AIR6	0.865			2.86	
AI perceived warmth	AIPW1	0.845	0.893	0.926	2.134	0.757
	AIPW2	0.891			2.82	
	AIPW3	0.875			2.553	
	AIPW4	0.867			2.373	
AI anthropomorphism	AIAn1	0.839	0.879	0.918	1.98	0.736
	AIAn2	0.861			2.302	
	AIAn3	0.865			2.399	
	AIAn4	0.867			2.351	
AI trust	AIT1	0.82	0.874	0.914	1.948	0.731
	AIT2	0.848			2.173	
	AIT3	0.88			2.517	
	AIT4	0.871			2.355	
AI attachment	AIA1	0.791	0.883	0.914	1.816	0.682
	AIA2	0.854			2.457	
	AIA3	0.865			2.58	
	AIA4	0.754			1.7	
	AIA5	0.859			2.448	
AI usage behavior	AIUB1	0.858	0.813	0.89	1.335	0.671
	AIUB2	0.73			2.432	
	AIUB3	0.739			2.256	
	AIUB4	0.744			2.039	

### 5.1 Convergent validity

This study employed PLS-SEM to examine the reliability and convergent validity of the measurement model, with the aim of assessing whether the constructed scales met acceptable standards in terms of internal consistency and construct validity. In evaluating measurement model validity, convergent validity, and discriminant validity, this study primarily followed the applicability and assessment criteria proposed by Cheng and Tsai (2020). The results indicate that the proposed model demonstrates satisfactory discriminant validity, with clear distinctions among latent constructs. Table 3 presents the Cronbach's α values, composite reliability (CR), and average variance extracted (AVE) for all latent variables. An AVE value greater than 0.50 is generally regarded as evidence of adequate convergent validity (Li, 2025). In the present study, the lowest AVE value was 0.671, which exceeds the recommended threshold of 0.50, indicating that all constructs exhibit satisfactory convergent validity. Furthermore, to confirm the internal consistency of the measurement scales, both Cronbach's α and CR were used to assess construct reliability. According to Hair et al. (2021b), Cronbach's α and CR values should range between 0 and 1 and exceed 0.70 to be considered acceptable. In this study, all constructs demonstrated Cronbach's α and CR values above the recommended threshold, indicating a high level of internal consistency and reliability. Consequently, the measurement model can be regarded as robust, lending strong support to the credibility of the research findings.

### 5.2 Model reliability and validity testing

According to Demler et al. (2015), the NFI values range from 0 to 1, with higher values indicating better model fit. In this study, the NFI value of 0.839 indicates an acceptable but moderate degree of fit, rather than a strong global fit. Although this value does not reach the more conservative baseline value of 0.90, commonly referred to in covariance-based SEM, it is within the acceptable range for PLS-SEM applications. Importantly, the standardized root mean square residual (SRMR) is 0.054, below the recommended threshold of 0.08, thus providing additional support for model adequacy. According to the predictive orientation of PLS-SEM, the global fit index is not considered the sole criterion for model evaluation; instead, the reliability, validity, and structural explanatory power of measurements are more emphasized. Overall, these results indicate that the model fit is sufficient and that the model exhibits moderate explanatory power; however, the extent of some structural effects remains limited. Please refer to Table 4 for specific details.

**Table 4:** Structural equation modeling (SEM) statistics.

Fit index	Saturated model	Estimated model
SRMR	0.054	0.062
NFI	0.839	0.833
d_G	0.972	1.032
d_ULS	2.154	2.812
Chi-square	3,10.491	3,109.854

To assess the discriminant validity of the measurement model, this study employed the heterotrait-monotrait ratio (HTMT) as the primary criterion, following the threshold recommended by Li (2025). According to this standard, HTMT values below 0.90 indicate adequate discriminant validity, suggesting that the latent constructs are empirically distinct from one another. SmartPLS was used to compute the HTMT values for all construct pairs, and the detailed results are presented in Table 5. The findings show that all HTMT values were below the recommended threshold of 0.90, with the highest value being 0.893. These results further confirm the satisfactory discriminant validity of the measurement model, indicating clear distinctions among the latent constructs. Taken together, the HTMT assessment provides robust evidence supporting the adequacy of discriminant validity in the proposed model, thereby confirming the model's rationality and credibility and providing a solid foundation for subsequent hypothesis testing and empirical analysis.

**Table 5:** Heterotrait-monotrait ratio (HTMT) Values.

Variable	AIA	AIPW	AIAn	AIR	AIT	AIUB
AIA						
AIPW	0.824					
AIAn	0.864	0.685				
AIR	0.731	0.747	0.654			
AIT	0.893	0.845	0.807	0.824		
AIUB	0.83	0.675	0.859	0.72	0.794	

At the same time, the Fornell-Lacker criterion was used to test discriminant validity. Table 6 shows that the square root values of the overall AVE of the latent variable are significantly larger than the correlation coefficients of the other determinants. In addition to applying the Fornell-Lacker test, this study also introduces the Heterotrait-Monotrait (HTMT) test method, which can be used to estimate the correlation coefficients of the latent variables. Through careful observation of the measured values, a high degree of discriminant validity between the measured indicators can be ensured.

**Table 6:** Fornell-Larcker criterion for discriminant validity.

Variable	AIA	AIPW	AIAn	AIR	AIT	AIUB
AIA	0.826					
AIPW	0.732	0.87				
AIAn	0.793	0.633	0.858			
AIR	0.662	0.678	0.616	0.852		
AIT	0.794	0.747	0.739	0.743	0.855	
AIUB	0.71	0.587	0.757	0.634	0.68	0.819

### 5.3 Model predictive ability

To evaluate the predictive ability of structural equation models (SEMs), this study adopted the coefficient of determination (R^2^) as an important indicator to measure the models' predictive ability. According to Shmueli et al. (2016), the coefficient of determination (R^2^) was calculated by the ratio of the sum of the squares of the regression to the sum of the total squares, reflecting the extent to which the predictors explained the variance of the dependent variable. In this study, the value of R^2^ is between 0.54 and 0.726, which shows that the model can effectively explain the variability of the dependent variable. In addition, f-squared values are often used in structural models to measure the contribution of changes in a latent variable to the predictive power of the dependent variable. There were often the following interpretation criteria: 0.02 < *f*^2^ < 0.15; 0.15 < *f*^2^ < 0.35; *f*^2^ > 0.35. Therefore, the effect of AIA on AIPW and AIR in this study, and the effect of AIPW on AIA, both show moderate or large effects, which indicates that these independent variables have strong explanatory power for the dependent variables. AIP has a smaller effect on AIUI, suggesting a weaker explanatory power of AIP for AIUI variables. Please refer to Table 7 for specific details.

**Table 7:** Coefficient of determination (R^2^) and Effect Size (f^2^).

*R^2^*		*f^2^*					
**AIA**	**0.725**	**AIA**	**AIPW**	**AIAn**	**AIR**	**AIT**	**AIUB**
AIPW							0.171
AIAn		0.175				0.166	
AIR		0.503				0.231	
AIT	0.726	0.03				0.179	0.079
AIUB	0.54						

To further validate the predictive ability of the study model, this study used the PLSPREDICT method for out-of-sample prediction evaluation, based on the suggestion of He and Ren (2025). According to the results in Table 8, the Q^2^ values for all endogenous variables ranged from 0.301 to 0.543. According to Shmueli et al. (2019), if PLS-SEM outperforms LM on a subset of metrics with *Q*^2^ > 0, it indicates that the predictive power of the model is moderate. Therefore, the structural model proposed in this study has moderate predictive power.

**Table 8:** Assessing the predictive power of models.

**Indicator**	*Q^2^*	PLS-SEM_RMSE	PLS-SEM_MAE	LM_RMSE	LM_MAE
AIA1	0.491	0.628	0.45	0.641	0.432
AIA2	0.52	0.711	0.495	0.729	0.497
AIA13	0.511	0.703	0.488	0.713	0.487
AIA14	0.378	0.767	0.548	0.781	0.546
AIA15	0.543	0.669	0.472	0.647	0.433
AIT1	0.486	0.719	0.516	0.733	0.506
AIT2	0.539	0.584	0.428	0.583	0.409
AIT3	0.535	0.582	0.411	0.583	0.411
AIT4	0.538	0.606	0.437	0.625	0.443
AIUB1	0.301	1.068	0.886	0.925	0.687
AIUB2	0.424	0.709	0.512	0.702	0.474
AIUB3	0.378	0.719	0.537	0.727	0.512
AIUB4	0.435	0.712	0.539	0.725	0.512

### 5.4 Hypothesis testing

**Figure 2:**
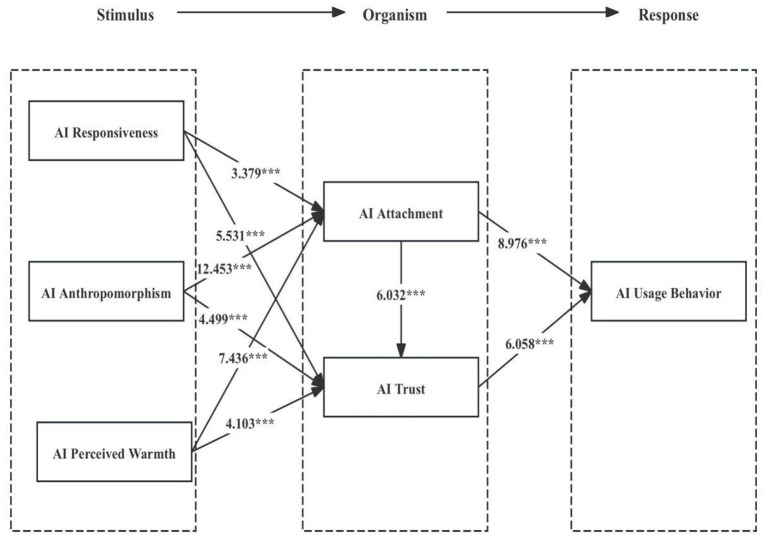
Structural equation path and significance. ***Indicate the correlation path coefficient *p* < 0.001.

Following the above validation procedures, SmartPLS (version 4.1) was employed to examine the direct effects, indirect effects, and total effects of the proposed structural equation model. Figure 2 presents the validated structural model, including the standardized path coefficients and their corresponding significance levels. A bootstrapping procedure with 5,000 resamples was conducted to estimate the variance, confidence intervals, *t-values*, and *p-values* for each structural path. The results of the data analysis and hypothesis testing are summarized in Table 9. The path coefficient from AI Anthropomorphism to AI attachment was statistically significant (β = 0.507, *p* < 0.001, SD = 0.041, *t* = 12.453), indicating that AI Anthropomorphism exerts a strong and positive effect on AI attachment. Similarly, the path from AI Anthropomorphism to AI trust was significant (β = 0.194, *p* < 0.001, SD = 0.043, *t* = 4.499), suggesting that higher levels of AI Anthropomorphism are associated with increased AI Trust. In addition, AI responsiveness demonstrated a significant positive influence on AI attachment (β = 0.132, *p* < 0.01, SD = 0.039, *t* = 3.379) as well as on AI Trust (β = 0.280, *p* < 0.001, SD = 0.051, *t* = 5.531). Likewise, AI perceived warmth significantly affected AI attachment (β = 0.322, *p* < 0.001, SD = 0.043, *t* = 7.436) and AI trust (β = 0.217, *p* < 0.001, SD = 0.053, *t* = 4.103). Furthermore, AI attachment exerted a significant positive effect on AI Trust (β = 0.297, *p* < 0.001, SD = 0.049, *t* = 6.032) and on AI usage behavior (β = 0.460, *p* < 0.001, SD = 0.051, *t* = 8.976). Finally, AI Trust also showed a significant positive effect on AI usage behavior (β = 0.314, *p* < 0.001, SD = 0.052, *t* = 6.058). Overall, the empirical findings provide strong support for the proposed research model, indicating that Hypotheses H1, H2, H3, H4, H5, H6, H7, H8, and H10 are all empirically supported.

**Table 9:** Path coefficients of the research framework.

Path name	β	SD	ConfidenceInterval		*t*	*p*	Significance	Decision
			2.50%	97.50%				
Direct impact								
AIA—AIT	0.297	0.049	0.2	0.394	6.032	0	< 0.001	Validated
AIA—AIUB	0.46	0.051	0.359	0.56	8.976	0	< 0.001	Validated
AIAn—AIA	0.507	0.041	0.429	0.587	12.453	0	< 0.001	Validated
AIAn—AIT	0.194	0.043	0.11	0.279	4.499	0	< 0.001	Validated
AIPW—AIA	0.322	0.043	0.238	0.407	7.436	0	< 0.001	Validated
AIPW—AIT	0.217	0.053	0.117	0.321	4.103	0	< 0.001	Validated
AIR—AIA	0.132	0.039	0.058	0.21	3.379	0.001	< 0.01	Validated
AIR—AIT	0.28	0.051	0.182	0.378	5.531	0	< 0.001	Validated
AIT—AIUB	0.314	0.052	0.21	0.412	6.058	0	< 0.001	Validated
Indirect impact								
AIA—AIT—AIUB	0.093	0.021	0.056	0.136	4.473	0	< 0.001	Validated
AIAn—AIT—AIUB	0.061	0.018	0.028	0.099	3.383	0.001	< 0.01	Validated
AIPW—AIT—AIUB	0.068	0.019	0.034	0.106	3.636	0	< 0.001	Validated
AIR—AIT—AIUB	0.088	0.023	0.047	0.139	3.764	0	< 0.001	Validated
AIAn—AIA—AIT	0.151	0.029	0.099	0.21	5.275	0	< 0.001	Validated
AIAn—AIA—AIUB	0.233	0.034	0.171	0.305	6.778	0	< 0.001	Validated
AIPW—AIA—AIT	0.096	0.021	0.058	0.139	4.644	0	< 0.001	Validated
AIPW—AIA—AIUB	0.148	0.024	0.103	0.197	6.206	0	< 0.001	Validated
AIR—AIA—AIT	0.039	0.013	0.016	0.067	2.987	0.003	< 0.01	Validated
AIR—AIA—AIUB	0.061	0.02	0.025	0.105	2.985	0.003	< 0.01	Validated
AIAn—AIA—AIT—AIUB	0.047	0.011	0.028	0.071	4.2	0	< 0.001	Validated
AIPW—AIA—AIT—AIUB	0.03	0.008	0.016	0.047	3.715	0	< 0.001	Validated
AIR—AIA—AIT—AIUB	0.012	0.004	0.005	0.022	2.85	0.004	< 0.01	Validated
Overall impact								
AIAn-AIUB	0.342	0.032	0.282	0.407	10.818	0	< 0.001	Validated
AIR-AIUB	0.161	0.031	0.103	0.224	5.143	0	< 0.001	Validated
AIPW-AIUB	0.096	0.021	0.188	0.304	8.389	0	< 0.001	Validated

Further examination of the data analysis results presented in Table 9 indicates that AI attachment plays a significant and positive mediating role in the relationships between AI anthropomorphism, AI perceived warmth, AI responsiveness, and AI usage behavior, respectively. At the same time, AI trust also serves as a significant positive mediator in the relationships between AI anthropomorphism, AI perceived warmth, AI responsiveness, and AI Usage Behavior. Accordingly, Hypotheses H9 and H11 are empirically supported. In addition, the results reveal significant chain mediation effects involving AI attachment and AI trust. Specifically, AI attachment and AI trust jointly form a significant positive serial mediation pathway between AI Anthropomorphism and AI usage behavior (β = 0.047, *p* < 0.001, SD = 0.011, *t* = 4.200). Similarly, AI attachment and AI trust exhibit a significant positive chain mediation effect between AI responsiveness and AI usage behavior (β = 0.012, *p* < 0.01, SD = 0.004, *t* = 2.850). Moreover, a significant positive serial mediation pathway is observed between AI perceived warmth and AI usage behavior through AI attachment and AI trust (β = 0.030, *p* < 0.001, SD = 0.008, *t* = 3.715). Taken together, these findings provide robust empirical support for the proposed chain mediation mechanisms, confirming that Hypotheses H12, H13, and H14 are all fully supported.

## 6 Discussion

### 6.1 AI anthropomorphism, attachment, and trust: distinct psychological pathways in conversational AI interaction

The results indicate that AI responsiveness positively predicts both AI attachment (β = 0.132, *p* < 0.01) and AI trust (β = 0.280, *p* < 0.001), thereby supporting H1 and H2. Timely feedback and task-oriented support appear to strengthen students' functional evaluations of conversational AI (Ng et al., 2024; Salimzadeh et al., 2024). Consistent with prior research, higher interactivity may reduce technological uncertainty and enhance interaction continuity, facilitating trust grounded in perceived system reliability (Fernandes and Oliveira, 2021; Guingrich and Graziano, 2023). In contrast, AI anthropomorphism shows the strongest effect on AI attachment (β = 0.507, *p* < 0.001), while exerting a smaller yet significant influence on AI trust (β = 0.194, *p* < 0.001), supporting H3 and H4. This pattern suggests that anthropomorphic cues may activate social heuristics, encouraging students to perceive AI as a relational “other” and shifting interaction from instrumental use toward emotionally meaningful engagement (Choudhury and Shamszare, 2023; Jin and Youn, 2023). Such findings align with social response perspectives in human-AI interaction, which propose that human-like language, emotional expression, and social presence can foster parasocial experiences and relational bonding (Polyportis and Pahos, 2025). Meanwhile, AI's perceived warmth significantly predicts both AI attachment (β = 0.322, *p* < 0.001) and AI trust (β = 0.217, *p* < 0.001), supporting H5 and H6, indicating that supportive interaction styles may enhance emotional safety and satisfaction, thereby strengthening relational engagement with AI systems (Huang and Gursoy, 2024; Liu et al., 2022; Deng and Yan, 2025).

First, the stronger effect of anthropomorphism on AI attachment suggests that emotional bonding is driven more by social-cognitive processing triggered by human-like cues than by functional performance alone. Prior research indicates that when technologies display social characteristics—such as conversational style, emotional expression, or interactional intent—users are more likely to categorize them as autonomous social actors, thereby shifting from tool-oriented processing to relationship-oriented processing (Wang et al., 2026). Anthropomorphic cues not only enhance engagement but also reduce perceived psychological distance, allowing users to incorporate AI into a social interaction framework and develop parasocial experiences. Unlike performance-based evaluations, this form of social processing emphasizes relational meaning rather than task efficiency, which may facilitate early attachment formation. Moreover, anthropomorphism may reshape attribution processes, encouraging users to interpret AI behavior in terms of intentions or social roles rather than algorithmic output. This shift from a functional object to a perceived social “other” provides a key theoretical basis for attachment development within student-conversational AI relationships. In contrast, the stronger association between responsiveness and AI trust indicates that trust judgments remain primarily grounded in evaluations of system capability and reliability. Research on automation trust suggests that consistent feedback and task performance function as diagnostic cues that reduce uncertainty and enhance predictability, thereby supporting competence-based trust formation (Ma et al., 2025). Within this framework, perceived warmth contributes to both attachment and trust, but mainly acts as an affective enhancer rather than a primary evaluative signal. Warmth-related cues increase emotional safety and interaction comfort, whereas capability and performance cues stabilize trust judgments (Song et al., 2022). Overall, conversational AI-supported learning reflects a dual interaction structure in which anthropomorphism facilitates emotional attachment, while responsiveness supports competence-based trust evaluation, highlighting distinct psychological pathways underlying human-AI interaction.

### 6.2 Affective-cognitive pathways to AI usage behavior: the distinct roles of AI attachment and AI trust

AI attachment was positively associated with university students' AI usage behavior, providing support for Hypothesis H7. Zhang et al. (2025) suggested that a stronger attachment enhances perceived convenience and relational closeness in digital interaction, thereby facilitating broader applications of conversational AI across contexts. Similarly, Rao Hill and Troshani (2024) found that frequent and high-intensity human-AI interaction is more likely to foster positive attachment rather than maladaptive technological dependence, which, in turn, promotes actual usage. Higher levels of AI attachment were also linked to stronger AI trust, supporting Hypothesis H8. Previous research has shown that attachment formed during interaction can enhance emotional experience and wellbeing, thereby reinforcing users' trust in AI systems (Ma et al., 2025). Wu (2024) further argued that stable and continuous interaction patterns, together with heightened emotional perception, can strengthen trust and increase the likelihood of sustained AI usage behavior. AI trust also showed a significant positive relationship with AI usage behavior, supporting Hypothesis H10. When students perceive AI systems as reliable and capable of providing accurate feedback, perceived risk and technology resistance tend to decrease, leading to improved learning efficacy and performance satisfaction (Kang, 2025). From a cognitive load perspective, well-designed AI systems can reduce information-processing demands and algorithm-induced overload, optimize learners' cognitive resource allocation, and ultimately facilitate sustained AIUB (Jose et al., 2025).

Although both AI attachment and AI trust significantly predicted AI usage behavior, the stronger effect of AI attachment (β = 0.460) compared with AI trust (β = 0.314) suggests that affective and cognitive pathways may play distinct roles in shaping students' behavioral engagement with conversational AI. AI attachment appears to operate primarily through an affective pathway, in which relational bonding, emotional resonance, and perceived companionship transform AI from a functional tool into a socially meaningful interaction partner. Such relational framing can generate intrinsic reinforcement, encouraging repeated and habitual interaction even in the absence of explicit task demands. Prior research on parasocial interaction and relational media use has similarly shown that emotional connection strengthens sustained engagement by enhancing perceived intimacy and relational satisfaction. In contrast, AI trust reflects a more cognitive pathway grounded in evaluations of reliability, predictability, and functional effectiveness (Munnukka et al., 2022). Trust reduces perceived risk and uncertainty, thereby enabling students to rely on AI during learning tasks. However, once a sufficient level of trust is established, its marginal contribution to increasing interaction frequency may diminish. This asymmetry helps explain why AI attachment demonstrated a stronger association with AI usage behavior: affective engagement directly energizes behavioral motivation, whereas cognitive trust primarily stabilizes usage decisions (Choudhury and Shamszare, 2023). From an educational perspective, these findings suggest that students' continued engagement with conversational AI may be driven less by purely rational assessments of system performance and more by relational experiences that foster ongoing interaction (Sheehan et al., 2020). At the same time, maintaining appropriate relational boundaries remains important, as excessive attachment could blur human-AI interaction limits and introduce potential psychosocial risks.

### 6.3 Dual mediation pathways in conversational AI engagement: differentiated roles of AI attachment and AI trust

In this study, AI attachment emerged as a key mediator linking AI responsiveness, AI anthropomorphism, and AI perceived warmth to AI usage behavior, suggesting that affective bonding plays a central role in transforming technological stimuli into sustained interaction. AI responsiveness, reflected in feedback accuracy and interaction efficiency, appears to provide a functional foundation for attachment development (Ferreira, 2024). By contrast, anthropomorphic cues and frequent interaction may shift students' perceptions of conversational AI from a functional tool to a socially meaningful “technological other,” allowing emotional connection to translate into greater engagement and usage (Jin and Youn, 2023). AI perceived warmth further strengthens social presence and emotional resonance, enhancing feelings of belonging and psychological support, which deepens attachment and promotes continued use (Nguyen and Le, 2025; Ma et al., 2025). Taken together, AI attachment reflects a transition from functional interaction toward relational engagement. AI trust also functioned as a cognitive mediator between technological cues and AI usage behavior. Consistent with trust theory, reliable and predictable interaction reduces perceived ethical risk and informational uncertainty, making students more willing to rely on conversational AI in learning contexts (Li et al., 2023). The humanlike linguistic capabilities enabled by large language models may further strengthen perceived ownership and interaction involvement, thereby enhancing trust and facilitating usage (Deng and Yan, 2025). In addition, warm and supportive feedback can increase psychological safety and wellbeing, which supports technology acceptance. Overall, AI attachment appears to energize interaction motivation through an affective pathway, whereas AI trust stabilizes adoption through cognitive evaluation, together forming a dual psychological mechanism underlying students' conversational AI usage behavior.

Comparisons of the mediation paths indicate that AI attachment and AI trust demonstrate differentiated mediation effects across distinct types of technological stimuli. Rather than reflecting a simple difference in strength, this pattern suggests that two psychological mechanisms are selectively responsive to different cues. In the anthropomorphism pathway, the indirect effect through AI attachment (AIP → AIA → AIUB, β = 0.233) was notably stronger than the pathway through AI trust (β = 0.061), implying that anthropomorphic signals are more likely to activate relational and social attribution processes. When students perceive conversational AI as a humanlike interaction partner, emotional bonding and parasocial tendencies are more readily formed, and these relational experiences translate into sustained use primarily through attachment rather than cognitive trust evaluation (Ta et al., 2020). A similar pattern emerged in the perceived warmth pathway, where AIPW → AIA → AIUB (β = 0.148) exceeded the trust-based indirect effect (β = 0.068). Warmth, supportive communication, and empathic responses appear to strengthen emotional engagement and a sense of belonging, thereby deepening attachment, whereas trust relies more heavily on judgments of reliability and stability and thus plays a secondary role under affective conditions (Pavone et al., 2023). By contrast, the responsiveness pathway showed an opposite tendency: AIR → AIT → AIUB (β = 0.088) was stronger than AIR → AIA → AIUB (β = 0.061), suggesting that performance- and efficiency-oriented cues promote usage behavior mainly by reducing uncertainty and perceived risk, which fosters trust rather than emotional bonding (Choudhury and Shamszare, 2023). Overall, these findings point to a differentiated mediation structure in which socio-emotional cues are more likely to operate through attachment, while functional performance cues are more likely to operate through trust. This dual-pathway mechanism provides a nuanced explanation of how university students psychologically engage with conversational AI systems.

### 6.4 The chain mediating effects of AI attachment and AI trust in conversational AI-assisted learning

The present study indicates that conversational AI responsiveness, anthropomorphism, and perceived warmth are indirectly associated with AI usage behavior through a sequential mediation of AI attachment and AI trust, providing statistical support for H12–H14. However, the magnitude of the chained effect was modest, suggesting that this pathway represents a limited contribution within the overall structural model. These findings are broadly consistent with prior research (Munnukka et al., 2022). From the perspective of the S-O-R framework, external technological cues may enhance users' perceptions of social presence and relational appraisal, which can be linked to attachment-related affective responses. Attachment, conceptualized as a relatively stable relational orientation developed through repeated interactions, may, in turn, facilitate the gradual formation of trust through attribution processes. In this sequence, attachment appears to stimulate interaction motivation, whereas trust may contribute to behavioral stability by reducing perceived uncertainty and risk. Rather than constituting a dominant explanatory mechanism, this progression can be interpreted as a complementary pathway in which affective engagement precedes cognitive evaluation. This interpretation is consistent with evidence suggesting that anthropomorphic cues and interactive communication styles are associated with increased social presence, attachment tendencies, and trust perceptions in conversational AI contexts (Deng and Yan, 2025).

From a broader educational perspective, recent applications of conversational AI teaching assistants in higher education provide contextual evidence that relational processes may play a role in shaping user engagement. When AI systems incorporate anthropomorphic expression and affective feedback, students may be more likely to perceive AI as socially responsive rather than purely instrumental. Such perceptions can be associated with relationally oriented psychological processing, through which technological cues are experienced as affectively meaningful interactions. Within this context, attachment may contribute to the development of trust, potentially influencing evaluations of system reliability and intentionality (Taneja et al., 2024). Psychologically, attachment and trust may operate at different stages: attachment can facilitate continued interaction through perceived social presence, whereas trust may stabilize behavioral decisions by reducing uncertainty and perceived risk. When these processes occur sequentially, user behavior may extend beyond short-term task engagement toward more sustained interaction patterns. However, given the marginal magnitude of the observed serial mediation effects, this two-stage interpretation should be understood as a limited explanatory pathway rather than a primary structural mechanism. Its practical influence on conversational AI usage appears comparatively small relative to other direct and parallel effects within the model (Chen et al., 2024).

## 7 Practical implications

Beyond demonstrating that conversational AI influences university students' learning behavior, the present study clarifies why and how these effects emerge, revealing that distinct technological cues operate through differentiated psychological pathways—AI attachment as an affective driver and AI trust as a cognitive stabilizer—thereby offering theoretically grounded directions for the design of intelligent educational systems.

The theoretical contributions of this study are threefold. First, AI anthropomorphism and AI perceived warmth function not merely as interface-level features, but as social attribution cues that reshape students' role perceptions of AI, transforming it from a functional tool into an interactive partner. This finding suggests that the value of conversational AI in educational contexts extends beyond efficiency gains, as social presence may strengthen the affective foundations of learning engagement and sustain ongoing interaction. Second, the mediation results indicate that socio-emotional cues, such as AI anthropomorphism and AI perceived warmth, are more likely to activate relational attribution and parasocial experiences, thereby promoting AI usage behavior primarily through attachment. This implies that sustained engagement is less driven by performance evaluation and more by intrinsic motivation derived from relational experience. Finally, the serial mediation mechanism reveals a differentiated psychological process in which attachment operates as an affective driver that initiates continuous interaction, whereas trust functions as a cognitive safeguard that reduces uncertainty and stabilizes adoption decisions. Together, these findings highlight the dual role of conversational AI in supporting both learning behavior and students' emotional and psychological wellbeing.

In terms of practical implications, this study offers several actionable insights. First, anthropomorphism and perceived warmth may prompt students to reframe AI from a functional tool into an interactive partner through social attribution processes. Therefore, universities should move beyond efficiency-oriented design and emphasize relational interaction features when implementing intelligent learning systems. Practical strategies include maintaining a consistent conversational persona, incorporating contextual memory and continuity in dialogue, and providing supportive responses in high-pressure learning situations to enhance social presence and sustain engagement. Second, because socio-emotional cues primarily influence usage behavior through AI attachment, educational platforms may integrate affective participation modules, such as periodic reflective dialogues, personalized encouragement, and low-threshold self-expression channels. These features can strengthen relational experiences and foster intrinsic motivation, shifting AI use from task-driven interaction toward habitual engagement. Finally, the serial mechanism suggests that attachment and trust serve distinct roles at different stages of behavioral formation. System design may therefore adopt a dual-layer structure that combines an “affective activation layer,” which promotes willingness to engage through empathic language and interaction continuity, with a “cognitive assurance layer,” which stabilizes adoption through source transparency, explainable feedback, and risk prompts. Overall, a design approach that initiates engagement through attachment while stabilizing decisions through trust may enhance learning participation while reducing the risk of overreliance, supporting the sustainable integration of conversational AI in higher education.

## 8 Limitation and future research directions

Due to practical constraints related to manpower, time, and research resources, this study is subject to several limitations, which also point to important directions for future research. The main limitations are as follows: First, methodological limitations should be acknowledged. This study primarily employed structural equation modeling (SEM) with a cross-sectional design to examine the chain mechanisms linking AI responsiveness, perceived warmth, AI anthropomorphism, AI attachment, AI trust, and usage behavior. However, the absence of qualitative data—such as in-depth interviews, observational data, or focus group discussions—limits a deeper understanding of students' emotional experiences and behavioral responses within AI-supported learning environments. Second, sample-related limitations exist. The research sample was drawn mainly from university students in Fujian Province, China. Although the sample demonstrates a certain level of representativeness, regional constraints related to cultural context, educational models, and AI usage behavior may restrict the generalizability and external validity of the findings. Third, variable selection limitations should be considered. This study focused on AI attachment and AI trust as key individual-level factors, while other potentially influential variables—such as AI literacy, mindfulness, loneliness, and related personality traits—were not included. These factors may also play important roles in shaping students' AI usage behaviors. Fourth, design limitations arise from the lack of a longitudinal approach. Owing to research constraints, this study did not adopt a longitudinal tracking design and therefore could not capture the dynamic and long-term evolution of students' emotional attachment, trust formation, and usage behaviors as their experience with conversational AI accumulates over time.

Based on these limitations, several directions for future research are proposed. First, future studies could adopt mixed-method research designs, integrating survey data with qualitative approaches such as in-depth interviews, to more comprehensively uncover the psychological dynamics and cognitive processes underlying AI usage behavior. Second, future research should expand the sampling scope to include students from different regions, types of higher education institutions, and academic disciplines, thereby enhancing representativeness and cross-cultural applicability. Third, incorporating additional psychological and contextual variables into more complex analytical models would allow for a more comprehensive explanation of the psychological-behavioral mechanisms operating in intelligent education environments. Such efforts would provide deeper insights into students' motivations, perceptions, and experiences before and after using intelligent learning tools. By addressing these issues, future research can offer a more systematic and in-depth exploration of how university students develop attachment and trust toward artificial intelligence and how these psychological processes ultimately translate into sustained use of conversational AI tools.

## 9 Conclusion

When university students use conversational AI-assisted learning tools, they are significantly influenced by external stimuli derived from the technological attributes of the system, including responsiveness, anthropomorphic features, and perceived warmth. These stimuli exert a substantial impact on students' emotional attachment to and trust in AI, thereby becoming critical antecedents shaping their learning-related usage behaviors. Moreover, AI attachment and AI trust play significant mediating roles in the relationships between responsiveness, Anthropomorphism, perceived warmth, and usage behavior. Beyond their individual effects, AI attachment and AI trust jointly constitute a salient chain mediation pathway characterized as “external stimuli–emotional perception–trust formation–usage behavior.” By introducing the Stimulus–Organism–Response (S-O-R) framework into the context of conversational AI–supported learning, this study extends beyond prevailing technology-centric and tool-oriented perspectives that have traditionally dominated research in this area. It expands the one-dimensional “technology–behavior” explanatory logic by systematically incorporating internal affective mechanisms—such as emotional attachment and psychological trust—into the analytical pathway. In doing so, the study innovatively constructs a comprehensive and multidimensional “technology–emotion–behavior” framework that captures the complexity, richness, and dual affective–cognitive nature of human–AI interaction in educational settings. This approach not only enriches and extends existing research but also provides an innovative and integrative framework for promoting healthy, sustainable, and well-balanced use of conversational AI-assisted learning tools among university students. Owing to its conceptual coherence, practical relevance, and broad applicability, employing the S-O-R framework to analyze university students' use of conversational AI for learning support carries substantial theoretical significance and practical value.

## Data Availability

The original contributions presented in the study are included in the article/Supplementary material, further inquiries can be directed to the corresponding author.

## References

[B1] Ajzen I. (1991). The theory of planned behavior. Organ. Behav. Hum. Decis. Process. 50, 179–211. doi: 10.1016/0749-5978(91)90020-T

[B2] Akpan I. J. Kobara Y. M. Owolabi J. Akpan A. A. Offodile O. F. (2025). Conversational and generative artificial intelligence and human–chatbot interaction in education and research. *Int. Trans. Oper*. Res. 32, 1251–1281. doi: 10.1111/itor.13522

[B3] Alagarsamy S. Mehrolia S. (2023). Exploring chatbot trust: antecedents and behavioural outcomes. Heliyon 9:e16074. doi: 10.1016/j.heliyon.2023.e16074 37206046 PMC10189503

[B4] Alam A. (2022). Social robots in education for long-term human–robot interaction: socially supportive behaviour of robotic tutor for creating robo-tangible learning environment in a guided discovery learning interaction. ECS Trans. 107, 12389–12403. doi: 10.1149/10701.12389ecst

[B5] Al-Oraini B. S. (2025). Chatbot dynamics: trust, social presence, and customer satisfaction in AI-driven services. J. Innov. Digit. Transform. 2, 109–130. doi: 10.1108/JIDT-08-2024-0022

[B6] Alqasir A. (2025). The relational shift: why we need “AI psychology” now as a core field. J. Psychol. AI 1:2573928. doi: 10.1080/29974100.2025.2573928

[B7] Anisha S. A. Sen A. Bain C. (2024). Evaluating the potential and pitfalls of AI-powered conversational agents as humanlike virtual health carers in the remote management of noncommunicable diseases: scoping review. *J. Med*. Internet Res. 26:e56114. doi: 10.2196/56114 PMC11289576 39012688

[B8] Apuke O. D. Omar B. Tunca E. A. Gever C. V. (2024). Information overload and misinformation sharing behaviour of social media users: testing the moderating role of cognitive ability. J. Inf. Sci. 50, 1371–1381. doi: 10.1177/01655515221121942

[B9] Bartneck C. Kulić D. Croft E. Zoghbi S. (2009). Measurement instruments for the anthropomorphism, animacy, likeability, perceived intelligence, and perceived safety of robots. Int. J. Soc. Robot. 1, 71–81. doi: 10.1007/s12369-008-0001-3

[B10] Bertacchini F. Demarco F. Scuro C. Pantano P. Bilotta E. (2023). A social robot connected with ChatGPT to improve cognitive functioning in ASD subjects. Front. Psychol. 14:1232177. doi: 10.3389/fpsyg.2023.1232177 37868599 PMC10585023

[B11] Bhattacherjee A. (2001). Understanding information systems continuance: an expectation-confirmation model. MIS Q. 25, 351–370. doi: 10.2307/3250921

[B12] Bradeško L. Mladenić D. (2012). “A survey of chatbot systems through a Loebner Prize competition,” in Proceedings of Slovenian Language Technologies Society Eighth Conference of Language Technologies (Ljubljana: Jožef Stefan Institute), 34–37.

[B13] Brennan K. A. Clark C. L. Shaver P. R. (1998). “Self-report measurement of adult attachment: an integrative overview,” in Attachment Theory and Close Relationships, eds. J. A. Simpson and W. S. *Rholes (New York, NY: Guilford Press)*, 46–76.

[B14] Carpinella C. M. Wyman A. B. Perez M. A. Stroessner S. J. (2017). “The robotic social attributes scale (RoSAS),” in 2017 12th ACM/IEEE International Conference on Human-Robot Interaction (HRI) (Vienna: IEEE), 254–262.

[B15] Chaturvedi R. Verma S. Das R. Dwivedi Y. K. (2023). Social companionship with artificial intelligence: recent trends and future avenues. *Technol. Forecast. Soc*. Change 193:122634. doi: 10.1016/j.techfore.2023.122634

[B16] Chen J. Guo F. Ren Z. Li M. Ham J. (2024). Effects of anthropomorphic design cues of chatbots on users' perception and visual behaviors. Int. J. Hum. Comput. Interact. 40, 3636–3654. doi: 10.1080/10447318.2023.2193514

[B17] Chen X. DiFranzo D. (2025). “Unpacking the dilemma: the dual impact of AI instructors' social presence on learners' perceived learning and satisfaction, mediated by the uncanny valley,” in Websci '25: 17th ACM Web Science Conference 2025 (New York, NY: ACM), 22–31.

[B18] Cheng K. H. Tsai C. C. (2020). Students' motivational beliefs and strategies, perceived immersion, and attitudes toward science learning with immersive virtual reality: a partial least squares analysis. Br. J. Educ. Technol. 51, 2140–2159. doi: 10.1111/bjet.12956

[B19] Chi N. T. K. Hoang V. N. (2023). Investigating customer trust in artificial intelligence: the role of anthropomorphism, empathy response, and interaction. CaaI Trans. Intell. Technol. 8, 260–273. doi: 10.1049/cit2.12133

[B20] Choudhury A. Shamszare H. (2023). Investigating the impact of user trust on the adoption and use of ChatGPT: survey analysis. J. Med. Internet Res. 25:e47184. doi: 10.2196/47184 37314848 PMC10337387

[B21] Cohen J. (1988). Statistical Power Analysis for the Behavioral Sciences, 2nd Edn. Hillsdale, NJ: Lawrence Erlbaum Associates.

[B22] Dang Q. Li G. (2025). Unveiling trust in AI: the interplay of antecedents, consequences, and cultural dynamics. AI Soc. 41, 669–692. doi: 10.1007/s00146-025-02477-6

[B23] Daud S. H. M. Teo N. H. I. Zain N. H. M. (2020). EJava chatbot for learning programming language: a post-pandemic alternative virtual tutor. Int. J. Emerg. Trends Eng. Res. 8, 3290–3298. doi: 10.30534/ijeter/2020/67872020

[B24] Davis F. D. (1989). Perceived usefulness, perceived ease of use, and user acceptance of information technology. MIS Q. 13, 319–340. doi: 10.2307/249008

[B25] Dehn D. M. Van Mulken S. (2000). The impact of animated interface agents: a review of empirical research. Int. J. Hum. Comput. Stud. 52, 1–22. doi: 10.1006/ijhc.1999.0325

[B26] Demler O. V. Paynter N. P. Cook N. R. (2015). Tests of calibration and goodness-of-fit in the survival setting. Stat. Med. 34, 1659–1680. doi: 10.1002/sim.6428 25684707 PMC4555993

[B27] Deng Z. Yan J. (2025). The effect of perceived warmth, competence, and social presence of AI-driven chatbots on consumers' engagement and satisfaction. SAGE Open 15:21582440251365438. doi: 10.1177/21582440251365438

[B28] Ding Y. Najaf M. (2024). Interactivity, humanness, and trust: a psychological approach to AI chatbot adoption in e-commerce. BMC Psychol. 12:595. doi: 10.1186/s40359-024-02083-z 39468563 PMC11514966

[B29] Donath L. Holenko-Dlab M. Mircea G. Muntean M. Neam?u M. Rozman T. . (2024). Perceptions' investigation regarding the need for upskilling in remote education: a PLS-SEM analysis. Econ. Comput. Econ. Cybern. Stud. Res. 58, 277–291. doi: 10.24818/18423264/58.3.24.17

[B30] Donnermann M. Schaper P. Lugrin B. (2025). Application of social robots in higher education: a long-term study. Int. J. Soc. Robot. 17, 2311–2326. doi: 10.1007/s12369-025-01286-7

[B31] Duong C. D. (2024). Modeling the determinants of higher education students' continuance intention to use ChatGPT for learning: a stimulus–organism–response approach. J. Res. Innov. Teach. Learn. 17, 391–407. doi: 10.1108/JRIT-01-2024-0006

[B32] Faul F. Erdfelder E. Buchner A. Lang A.-G. (2009). Statistical power analyses using G^*^Power 3.1: tests for correlation and regression analyses. Behav. Res. Methods 41, 1149–1160. doi: 10.3758/BRM.41.4.1149 19897823

[B33] Feng H. Mahoor M. H. Dino F. (2022). A music-therapy robotic platform for children with autism: a pilot study. Front. Robot. AI 9:855819. doi: 10.3389/frobt.2022.855819 35677082 PMC9169087

[B34] Fernandes T. Oliveira E. (2021). Understanding consumers' acceptance of automated technologies in service encounters: drivers of digital voice assistants adoption. J. Bus. Res. 122, 180–191. doi: 10.1016/j.jbusres.2020.08.058

[B35] Ferreira J. F. N. (2024). Exploring the dynamics of trust in recommendation chatbots: the roles of perceived value, parasocial interaction, and anthropomorphism. [Doctoral Dissertation]. ISCTE–Instituto Universitário de Lisboa, Portugal.

[B36] Fink M. C. Robinson S. A. Ertl B. (2024). AI-based avatars are changing the way we learn and teach: benefits and challenges. Front. Educ. 9:1416307. doi: 10.3389/feduc.2024.1416307

[B37] Flavián C. Casaló L. V. (2021). Artificial intelligence in services: current trends, benefits and challenges. Serv. Ind. J. 41, 853–859. doi: 10.1080/02642069.2021.1989177

[B38] Go E. Sundar S. S. (2019). Humanizing chatbots: the effects of visual, identity and conversational cues on humanness perceptions. Comput. Hum. Behav. 97, 304–316. doi: 10.1016/j.chb.2019.01.020

[B39] Guingrich R. E. Graziano M. S. A. (2023). Chatbots as social companions: how people perceive consciousness, human likeness, and social health benefits in machines. arXiv [Preprint]. *arXiv.2311.10599*. doi: 10.48550/arXiv.2311.10599

[B40] Hair J. F. Hult G. T. M. Ringle C. M. Sarstedt M. (2021a). A Primer on Partial Least Squares Structural Equation Modeling (PLS-SEM), 3rd Edn. Thousand Oaks, CA: Sage Publications.

[B41] Hair J. F. Hult G. T. M. Ringle C. M. Sarstedt M. Danks N. P. Ray S. (2021b). Partial Least Squares Structural Equation Modeling (PLS-SEM) Using R: A Workbook. Cham: Springer Nature.

[B42] Hair J. F. Ringle C. M. Gudergan S. P. Fischer A. Nitzl C. Menictas C. (2019). Partial least squares structural equation modeling-based discrete choice modeling: an illustration in modeling retailer choice. Bus. Res. 12, 115–142. doi: 10.1007/s40685-018-0072-4

[B43] Haman M. Školník M. Kučírková K. (2024). The rise of talking machines: balancing the potential and pitfalls of voice chatbots for mental wellbeing. J. Public Health 46, e715–e716. doi: 10.1093/pubmed/fdae269 39354652

[B44] He S. Ren Y. (2025). Exploring pre-service music teachers' acceptance of generative artificial intelligence: a PLS-SEM–ANN approach. Front. Psychol. 16:1571279. doi: 10.3389/fpsyg.2025.1571279 40657578 PMC12247848

[B45] Heng Z. Liu Y. Jiang M. Chen J. Wang M. Pass F. (2025). Emotional artificial intelligence in education: a systematic review and meta-analysis. Educ. Psychol. Rev. 37:10086. doi: 10.1007/s10648-025-10086-4

[B46] Hochreiter V. Benedetto C. Loesch M. (2023). The stimulus–organism–response (S-O-R) paradigm as a guiding principle in environmental psychology: comparison of its usage in consumer behavior and organizational culture and leadership theory. J. Entrep. Bus. Dev. 3, 7–16. doi: 10.18775/jebd.31.5001

[B47] Hoff K. A. Bashir M. (2015). Trust in automation: integrating empirical evidence on factors that influence trust. Hum. Factors 57, 407–434. doi: 10.1177/0018720814547570 25875432

[B48] Hu Q. Lu Y. Pan Z. Gong Y. Yang Z. (2021). Can AI artifacts influence human cognition? The effects of artificial autonomy in intelligent personal assistants. Int. J. Inf. Manag. 56:102250. doi: 10.1016/j.ijinfomgt.2020.102250

[B49] Huang T. (2023). Using the S-O-R framework to explore the driving factors of older adults' smartphone use behavior. *Humanit. Soc. Sci*. Commun. 10:690. doi: 10.1057/s41599-023-02221-9

[B50] Huang Y. Gursoy D. (2024). Customers' online service encounter satisfaction with chatbots: interaction effects of language style and decision-making journey stage. Int. J. Contemp. Hosp. Manag. 36, 4074–4091. doi: 10.1108/IJCHM-11-2023-1800

[B51] Huo C. Wang X. Sadiq M. W. Pang M. (2023). Exploring factors affecting consumers' impulse buying behavior in live-streaming shopping: an interactive research based upon the S-O-R model. SAGE Open 13:21582440231172678. doi: 10.1177/21582440231172678

[B52] Jacoby J. (2002). Stimulus–organism–response reconsidered: an evolutionary step in modeling (consumer) behavior. J. Consum. Psychol. 12, 51–57. doi: 10.1207/S15327663JCP1201_05

[B53] Jia C. Li P. (2024). Generation Z's health information avoidance behavior: insights from focus group discussions. J. Med. Internet Res. 26:e54107. doi: 10.2196/54107 38457223 PMC10960220

[B54] Jin S. V. Youn S. (2023). Social presence and imagery processing as predictors of chatbot continuance intention in human–AI interaction. Int. J. Hum. Comput. Interact. 39, 1874–1886. doi: 10.1080/10447318.2022.2129277

[B55] Jose B. Cherian J. Verghis A. M. Varghise S. M. Mumthas S. Joseph S. (2025). The cognitive paradox of AI in education: between enhancement and erosion. Front. Psychol. 16:1550621. doi: 10.3389/fpsyg.2025.1550621 40297599 PMC12036037

[B56] Kang W. Shao B. Zhang Y. (2024). How does interactivity shape users' continuance intention of intelligent voice assistants? Evidence from SEM and fsQCA. Psychol. Res. Behav. Manag. 17, 867–889. doi: 10.2147/PRBM.S438465 PMC10933529 38481599

[B57] Kang J. M. (2025). The impact of trust in AI shopping chatbots on flow and continued usage intention: comparing Republic of Korea and China. Authorea [Preprint]. doi: 10.31124/advance.174773431.13141953/v1

[B58] Kim Y. Sundar S. S. (2012). Anthropomorphism of computers: is it mindful or mindless? Comput. Hum. Behav. 28, 241–250. doi: 10.1016/j.chb.2011.09.006

[B59] Klaus P. Zaichkowsky J. (2020). AI voice bots: a services marketing research agenda. J. Serv. Mark. 34, 389–398. doi: 10.1108/JSM-01-2019-0043

[B60] Kocdar S. (2017). Book review: Designing teaching and learning for a digital age. Int. Rev. Res. Open Distrib. Learn. 18:159–162. doi: 10.19173/irrodl.v18i3.3107

[B61] Kock N. (2015). Common method bias in PLS-SEM: a full collinearity assessment approach. Int. J. E-Collab. 11, 1–10. doi: 10.4018/ijec.2015100101

[B62] Konadu B. O. Kusi E. (2025). AI chatbots and students' mental health support: an efficacy review. Am. J. Educ. Learn. 10, 207–225. doi: 10.55284/ajel.v10i2.1554

[B63] Kyrlitsias C. Michael-Grigoriou D. (2022). Social interaction with agents and avatars in immersive virtual environments: a survey. *Front*. Virtual Real. 2:786665. doi: 10.3389/frvir.2021.786665

[B64] Laban G. Wang J. Gunes H. (2025). A robot-led intervention for emotion regulation: from expression to reappraisal. arXiv [Preprint]. *arXiv.2503.18243*. doi: 10.48550/arXiv.2503.18243

[B65] Lee Y. K. Jung Y. Kang G. Hahn S. (2023). Developing social robots with empathetic non-verbal cues using large language models. arXiv [Preprint]. *arXiv.2308.16529*. doi: 10.48550/arXiv.2308.16529

[B66] Li Q. Luximon Y. Zhang J. (2023). The influence of anthropomorphic cues on patients' perceived anthropomorphism, social presence, trust building, and acceptance of healthcare conversational agents: a within-subject web-based experiment. *J. Med*. Internet Res. 25:e44479. doi: 10.2196/44479 PMC10450539 37561567

[B67] Li W. (2025). A study on factors influencing designers' behavioral intention in using AI-generated content for assisted design: perceived anxiety, perceived risk, and UTAUT. Int. J. Hum. Comput. Interact. 41, 1064–1077. doi: 10.1080/10447318.2024.2310354

[B68] Liu C.-C. Liao M.-G. Chang C.-H. Lin H.-M. (2022). An analysis of children's interaction with an AI chatbot and its impact on their interest in reading. Comput. Educ. 189:104576. doi: 10.1016/j.compedu.2022.104576

[B69] Liu Y. (2003). Developing a scale to measure the interactivity of websites. J. Advert. Res. 43, 207–216. doi: 10.2501/jar-43-2-207-216

[B70] Liu Y. Liu Y. (2025). Advancing STEM education for sustainability: the impact of graphical knowledge visualization and user experience on continuance intention in mixed-reality environments. Sustainability 17:3869. doi: 10.3390/su17093869

[B71] Ma N. Khynevych R. Hao Y. Wang Y. (2025). Effect of anthropomorphism and perceived intelligence in chatbot avatars of visual design on user experience: accounting for perceived empathy and trust. *Front. Comput*. Sci. 7:1531976. doi: 10.3389/fcomp.2025.1531976

[B72] Malakcioglu C. (2024). Emotional loneliness, perceived stress, and academic burnout of medical students after the COVID-19 pandemic. Front. Psychol. 15:1370845. doi: 10.3389/fpsyg.2024.1370845 39108428 PMC11301781

[B73] McKee K. R. Bai X. Fiske S. T. (2023). Humans perceive warmth and competence in artificial intelligence. iScience 26:107256. doi: 10.1016/j.isci.2023.107256 37520710 PMC10371826

[B74] McKnight D. H. Cummings L. L. Chervany N. L. (1998). Initial trust formation in new organizational relationships. Acad. Manage. Rev. 23, 473–490. doi: 10.2307/259290

[B75] Mehrabian A. Russell J. A. (1974). An Approach to Environmental Psychology. Cambridge, MA: MIT Press.

[B76] Mieczkowski H. Liu S. X. Hancock J. Reeves B. (2019). “Helping not hurting: applying the stereotype content model and BIAS map to social robotics,” in https://ieeexplore.ieee.org/xpl/conhome/8666012/proceeding 2019 14th ACM/IEEE International Conference on Human-Robot Interaction (HRI) (Daegu: IEEE), 222–229.

[B77] Munnukka J. Talvitie-Lamberg K. Maity D. (2022). Anthropomorphism and social presence in human–virtual service assistant interactions: the role of dialog length and attitudes. *Comput. Hum*. Behav. 135:107343. doi: 10.1016/j.chb.2022.107343

[B78] Ng D. T. K. Tan C. W. Leung J. K. L. (2024). Empowering student self-regulated learning and science education through ChatGPT: a pioneering pilot study. Br. J. Educ. Technol. 55, 1328–1353. doi: 10.1111/bjet.13454

[B79] Nguyen L. A. D. Le T. T. P. (2025). Exploring the effects of an AI chatbot on emotional engagement in English speaking lessons: insights from Call Annie. Int. J. AI Lang. Educ. 2, 79–99. doi: 10.54855/ijaile.25225

[B80] Nikolov A. N. Iyer P. Rokonuzzaman M. . (2025). The AI chatbot anthropomorphism dilemma. *Mark. Intell*. Plan. 43, 1–24. doi: 10.1108/MIP-09-2024-0623

[B81] Pavone G. Meyer-Waarden L. Munzel A. (2023). Rage against the machine: experimental insights into customers' negative emotional responses, attributions of responsibility, and coping strategies in artificial intelligence–based service failures. J. Interact. Mark. 58, 52–71. doi: 10.1177/10949968221134492

[B82] Peng M. Y. P. Xu Y. Xu C. (2023). Enhancing students' English language learning via mobile learning: integrating the technology acceptance model and the S-O-R model. Heliyon 9:e13447. doi: 10.1016/j.heliyon.2023.e13302 36755609 PMC9900360

[B83] Pitts G. Motamedi S. (2025). Understanding human–AI trust in education. arXiv [Preprint]. *arXiv.2506.09160*. doi: 10.48550/arXiv.2506.09160

[B84] Polyportis A. Pahos N. (2025). Understanding students' adoption of the ChatGPT chatbot in higher education: the role of anthropomorphism, trust, design novelty, and institutional policy. Behav. Inf. Technol. 44, 315–336. doi: 10.1080/0144929X.2024.2317364

[B85] Qiu L. Benbasat I. (2009). Evaluating anthropomorphic product recommendation agents: a social relationship perspective to designing information systems. Int. J. Hum. Comput. Stud. 67, 689–711. doi: 10.2753/MIS0742-1222250405

[B86] Rao Hill S. Troshani I. (2024). Chatbot anthropomorphism, social presence, uncanniness and brand attitude effects. *J. Comput. Inf* . Syst. 64, 1–17. doi: 10.1080/08874417.2024.2423187

[B87] Reeves B. Hancock J. Liu X. (2020). Social robots are like real people: first impressions, attributes, and stereotyping of social robots. Technol. Mind Behav. 1, 76–89. doi: 10.1037/tmb0000018

[B88] Reyes-Portillo J. A. So A. McAlister K. Nicodemus C. Golden A. Jacobson C. . (2025). Generative AI–powered mental wellness chatbot for college student mental wellness: open trial. JMIR Form. Res. 9:e71923. doi: 10.2196/71923 40726405 PMC12303582

[B89] Romero-Charneco M. Casado-Molina A. M. Alarcón-Urbistondo P. Cabrera Sánchez J. P. (2025). Customer intentions toward the adoption of WhatsApp chatbots for restaurant recommendations. J. Hosp. Tour. Technol. 16, 784–816. doi: 10.1108/JHTT-01-2024-0024

[B90] Salimzadeh S. He G. Gadiraju U. (2024). “Dealing with uncertainty: understanding the impact of prognostic versus diagnostic tasks on trust and reliance in human–AI decision making,” in CHI Conference on Human Factors in Computing Systems (Honolulu, HI: ACM), 1–17.

[B91] Salloum S. A. Alomari K. M. Alfaisal A. M. Aljanada R. A. Basiouni A. (2025). Emotion recognition for enhanced learning: using AI to detect students' emotions and adjust teaching methods. Smart Learn. Environ. 12:21. doi: 10.1186/s40561-025-00374-5

[B92] Sarstedt M. Hair J. F. Pick M. Liengaard B. D. Radomir L. Ringle C. M. (2022). Progress in partial least squares structural equation modeling use in marketing research in the last decade. Psychol. Mark. 39, 1035–1064. doi: 10.1002/mar.21640

[B93] Schouten D. G. M. Deneka A. A. Theune M. Neerincx M. A. Cremers A. H. M. (2023). An embodied conversational agent coach to support societal participation learning by low-literate users. Univ. Access Inf. Soc. 22, 1215–1241. doi: 10.1007/s10209-021-00865-5

[B94] Seitz L. (2024). Artificial empathy in healthcare chatbots: does it feel authentic? Comput. Hum. Behav. Artif. Hum. 2:100067. doi: 10.1016/j.chbah.2024.100067

[B95] Seo S. (2022). When female (male) robot is talking to me: effect of service robots' gender and anthropomorphism on customer satisfaction. *Int. J. Hosp*. Manag. 102:103166. doi: 10.1016/j.ijhm.2022.103166

[B96] Shahzad M. F. Xu S. An X. Javed I. (2024). Assessing the impact of AI-chatbot service quality on user e-brand loyalty through chatbot user trust, experience, and electronic word of mouth. *J. Retail. Consum*. Serv. 79:103867. doi: 10.1016/j.jretconser.2024.103867

[B97] Sharpe P. Ciriello R. F. (2024). “Exploring attachment and trust in AI companion use,” in Exploring Attachment and Trust in AI Companion Use (ACIS 2024) (Canberra, ACT: Association for Information Systems), 49.

[B98] Sheehan B. Jin H. S. Gottlieb U. (2020). Customer service chatbots: anthropomorphism and adoption. J. Bus. Res. 115, 14–24. doi: 10.1016/j.jbusres.2020.04.030

[B99] Shi X. Evans R. Shan W. (2022). Solver engagement in online crowdsourcing communities: the roles of perceived interactivity, relationship quality, and psychological ownership. *Technol. Forecast. Soc*. Change 175:121389. doi: 10.1016/j.techfore.2021.121389

[B100] Shin D. (2021). The effects of explainability and causability on perception, trust, and acceptance: implications for explainable AI. Int. J. Hum. Comput. Stud. 146:102551. doi: 10.1016/j.ijhcs.2020.102551

[B101] Shmueli G. Ray S. Estrada J. M. V. Chatla S. B. (2019). Predictive model assessment in PLS-SEM: guidelines for using PLSpredict. Eur. J. Mark. 53, 2322–2347. doi: 10.1108/EJM-02-2019-0189

[B102] Shmueli G. Ray S. Estrada J. M. V. Chatla S. B. (2016). The elephant in the room: predictive performance of PLS models. J. Bus. Res. 69, 4552–4564. doi: 10.1016/j.jbusres.2016.03.049

[B103] Shu C. Lai K. L. He L. (2026). Human–AI attachment: how humans develop intimate relationships with AI. Front. Psychol. 17:1723503. doi: 10.3389/fpsyg.2026.1723503 41756487 PMC12932595

[B104] Siyou L. Wan Husin W. N. I. B. W. (2025). The mediating role of loneliness in the relationship between academic burnout and psychological well-being. Int. J. Acad. Res. Prog. Educ. Dev. 14, 1940–1963. doi: 10.6007/IJARPED/v14-i2/25634

[B105] Skjuve M. Følstad A. Fostervold K. I. Brandtzaeg P. B. (2021). My chatbot companion: a study of human–chatbot relationships. Int. J. Hum.–Comput. Stud. 149:102601. doi: 10.1016/j.ijhcs.2021.102601

[B106] Song M. Xing X. Duan Y. Cohen J. Mou J. (2022). Will artificial intelligence replace human customer service? The impact of communication quality and privacy risks on adoption intention. J. Retail. Consum. Serv. 66:102900. doi: 10.1016/j.jretconser.2021.102900

[B107] Ta V. Griffith C. Boatfield C. Wang X. Civitello M. Bader H. . (2020). User experiences of social support from companion chatbots in everyday contexts: thematic analysis. J. Med. Internet Res. 22:e16235. doi: 10.2196/16235 32141837 PMC7084290

[B108] Tan L. Y. Hu S. Yeo D. J. Cheong K. H. (2025). Artificial intelligence-enabled adaptive learning platforms: a review. *Comput. Educ. Artif* . Intell. 6:100429. doi: 10.1016/j.caeai.2025.100429

[B109] Taneja K. Maiti P. Kakar S. Guruprasad P. Rao S. Goel A. K. (2024). “Jill Watson: a virtual teaching assistant powered by ChatGPT,” in Artificial Intelligence in Education. AIED 2024. Lecture Notes in Computer Science, eds. A. M. Olney, I. A. Chounta, Z. Liu, O. C. Santos, and I. I. Bittencourt (Cham: Springer), 324–337.

[B110] Vafaei-Zadeh A. Nikbin D. Wong S. L. Hanifah H. (2025). Investigating factors influencing AI customer service adoption: an integrated model of stimulus–organism–response (S-O-R) and task–technology fit (TTF) theory. Asia Pac. J. Mark. Logist. 37, 1465–1502. doi: 10.1108/APJML-05-2024-0570

[B111] Venkatesh V. Morris M. G. Davis G. B. Davis F. D. (2003). User acceptance of information technology: toward a unified view. MIS Q. 27, 425–478. doi: 10.2307/30036540

[B112] Veras M. Labbé D. R. Furlano J. Zakus D. Rutherford D. Pendergast B. . (2023). A framework for equitable virtual rehabilitation in the metaverse era: challenges and opportunities. Front. Rehabil. Sci. 4:1241020. doi: 10.3389/fresc.2023.1241020 37691912 PMC10488814

[B113] Wang J. Wen S. Xue J. (2023). Unraveling the influential mechanisms of social commerce overloads on user disengagement: the buffer effect of guanxi. Psychol. Res. Behav. Manag. 16, 1921–1945. doi: 10.2147/PRBM.S408119 37260935 PMC10228529

[B114] Wang S. Chen C. C. (2024). Exploring designer trust in artificial intelligence-generated content: a TAM/TPB model study. Appl. Sci. 14:6902. doi: 10.3390/app14166902

[B115] Wang S. Fatima N. Shahbaz M. Asif M. (2026). Building user trust in AI chatbots for customer service through human-like cues and perceived reliability. Sci. Rep. 16:38179. doi: 10.1038/s41598-026-38179-2 41663521 PMC12953761

[B116] Waytz A. Heafner J. Epley N. (2014). The mind in the machine: anthropomorphism increases trust in an autonomous vehicle. *J. Exp. Soc*. Psychol. 52, 113–117. doi: 10.1016/j.jesp.2014.01.005

[B117] Weizenbaum J. (1966). ELIZA—a computer program for the study of natural language communication between man and machine. Commun. ACM 9, 36–45. doi: 10.1145/365153.365168

[B118] Wik P. Hjalmarsson A. (2009). Embodied conversational agents in computer-assisted language learning. Speech Commun. 51, 1024–1037. doi: 10.1016/j.specom.2009.05.006

[B119] Winkler R. Söllner M. (2018). Unleashing the potential of chatbots in education: a state-of-the-art analysis. Acad. Manag. Proc. 2018:15903. doi: 10.5465/AMBPP.2018.15903abstract

[B120] Wu J. (2024). Social and ethical impact of emotional AI advancement: the rise of pseudo-intimacy relationships and challenges in human interactions. Front. Psychol. 15:1410462. doi: 10.3389/fpsyg.2024.1410462 39564589 PMC11573535

[B121] Wut T. M. Lee S. W. Xu J. Kwok M. L. J. (2025). Do trusting belief and social presence matter? Service satisfaction in using AI chatbots: necessary condition analysis and importance–performance map analysis. Informatics 12:91. doi: 10.3390/informatics12030091

[B122] Yang F. Oshio A. (2025). Using attachment theory to conceptualize and measure the experiences in human–AI relationships. Curr. Psychol. 44, 10658–10669. doi: 10.1007/s12144-025-07917-6

[B123] Yang J. Peng M. Y. P. Wong S. H. Chong W. (2021). How e-learning environmental stimuli influence determinants of learning engagement in the context of COVID-19? An S-O-R model perspective. Front. Psychol. 12:584976. doi: 10.3389/fpsyg.2021.584976 33868072 PMC8044515

[B124] Yaseen H. Mohammad A. S. Ashal N. Abusaimeh H. Ali A. Sharabati A.-A. A. (2025). The impact of adaptive learning technologies, personalized feedback, and interactive AI tools on student engagement: the moderating role of digital literacy. Sustainability 17:1133. doi: 10.3390/su17031133

[B125] Youn S. Jin S. V. (2021). In AI we trust? The effects of parasocial interaction and technopian versus luddite ideological views on chatbot-based customer relationship management in the emerging “feeling economy.” *Comput. Hum. Behav*. 119:106721. doi: 10.1016/j.chb.2021.106721

[B126] Zhang K. Xie Y. Chen D. Ji Z. Wang J. (2025). Effects of attractions and social attributes on people's usage intention and media dependence towards chatbots: the mediating role of parasocial interaction and emotional support. BMC Psychol. 13:986. doi: 10.1186/s40359-025-03284-w 40883845 PMC12398025

[B127] Zhang R. W. Liang X. Wu S.-H. (2024). When chatbots fail: exploring user coping following chatbot-induced service failure. Inf. Technol. People 37, 175–195. doi: 10.1108/ITP-08-2023-0745

[B128] Zhang X. Chen A. L. Piao X. Yu M. Zhang Y. Zhang L. (2024). Is AI chatbot recommendation convincing customers? An analytical response based on the elaboration likelihood model. Acta Psychol. 250:104501. doi: 10.1016/j.actpsy.2024.104501 39357416

